# Supplementary material for: Winter is coming: Pathogen emergence in seasonal environments
Source: PLoS Comput Biol. 2020 Jul 6;16(7):e1007954. doi: 10.1371/journal.pcbi.1007954 (PMC7365480; doi:10.1371/journal.pcbi.1007954)
Supplement: S1 Text — We present: (1) a calculation of the probability of emergence of directly transmitted pathogen for different scenarios of seasonality, (2) a generalisation of our results when the pathogen life cycle goes through multiple stages before completing its life cycle, (3) an exploration of the winter is coming effect on the seasonal dynamics of Zika virus, (4) an analysis of a scenario that involves pulse interventions (vaccination or treatment), (5) an exploration of the effect of density dependence on the winter is coming effect, (6) additional computations and proofs. (PDF) [file pcbi.1007954.s001.pdf]

WINTER IS COMING: PATHOGEN EMERGENCE IN SEASONAL ENVIRONMENTS

- SUPPLEMENTARY INFORMATION -

PHILIPPE CARMONA AND SYLVAIN GANDON

Contents

1. **Case studies** 2
   1.1. Square wave 2
      1.1.1. Control strategies 2
   1.2. Sinusoidal wave 4
   1.3. When the time of introduction is not uniformly distributed 5
2. **Pathogen emergence for time varying multitype birth and death processes** 6
   2.1. An ODE satisfied by the extinction probabilities 6
   2.2. A special case: when all rates are constant 9
      In dimension $d = 1$ 9
      In dimension $d = 2$ 9
      The *cyclic* case 9
      Effect of the type of the first infected host on p_e 10
   2.3. The numerical approach of Bacaër and Ait Dads 10
   2.4. Asymptotic results for large periods: $T \to +\infty$ 13
   2.5. A direct proof of Kendall's formula for the emergence probability 14
3. **A vector borne disease : Zika virus** 15
   Model I 16
   Model II 18
4. **Pulse interventions** 19
   4.1. A pulse vaccination model 19
   4.2. A pulse treatment model 22
5. **A density dependent example** 24
6. **Additional computations and proofs** 28
   6.1. Proof of Proposition 6.1 28
   6.2. Proof of Proposition 1.1. 29
References 35

1. Case studies

1.1. **Square wave.** We assume that death rate is constant $\mu(t) = 1$ and $\lambda(t) = \lambda_0 \ \mathbf{1}_{(0<t<1-\gamma)}$ with $\lambda_0 > 1$. Assume furthermore that $R_0 > 1$ that is $\bar{\lambda} > \bar{\mu}$ with $\bar{\lambda} = \int_0^1 \lambda(s)\,ds = \lambda_0(1-\gamma)$ and $\bar{\mu} = 1$.
The limiting probability of emergence is

$$p_{e,\infty}(t_0) = (1 - \frac{1}{\lambda_0})\,\mathbf{1}_{(0<t_0<t^*)}\,, \quad \text{with} \quad t^* = \frac{\bar{\lambda}-1}{\lambda_0 - 1} = \frac{\varphi(1)}{\lambda_0 - 1} \in (0, 1-\gamma)\,. \quad (1.1)$$

Indeed, the function φ is the 1 periodic function corresponding to the hat function of Figure A, and t^* is the only solution in $(0,1)$ of $\varphi(t_0) = \varphi(1)$: for $t_0 \in (t^*, 1)$ there exists $s > 0$ such that $\varphi(s+t_0) < \varphi(t_0)$ and for $t \in (0, t^*)$, for any $s > 0$, $\varphi(s+t_0) > \varphi(t_0)$.

1.1.1. *Control strategies.* We consider control strategies that lower the birth rate $\lambda(t)$ by a factor $\rho(t)$. More precisely we replace $\lambda(t)$ by

$$\lambda_\rho(t) = \lambda(t)(1-\rho(t)) \quad (1.2)$$

with $\rho(t) \in [0,1]$. Accordingly $\varphi(t)$ is replaced by $\varphi_\rho(t) = \varphi(t) - \int_0^t \lambda(s)\rho(s)\,ds$ and the probability of emergence $p_{e,\infty,\rho}(t_0)$ is decreased. We measure the quality of the control strategy by averaging this quantity

$$\langle p_{e,\infty,\rho}\rangle := \int_0^1 p_{e,\infty,\rho}(t_0)\,dt_0\,. \quad (1.3)$$

Indeed $\langle p_{e,\infty,\rho}\rangle$ is the mean probability of emergence for an infected who arrives uniformly in the period.
The *cost* of the control strategy is

$$C(\rho) := \int_0^1 \rho(s)ds\,. \quad (1.4)$$

We are looking for strategies with a given cost $C = C(\rho)$ which minimize $\langle p_{e,\infty,\rho}\rangle$. We shall assume from now on thath the control strategies are of the type

$$\rho(t) = \rho_M\,\mathbf{1}_{(t_1<t<t_2)} \quad (1.5)$$

with $0 \le \rho_M \le 1$ and $0 \le t_1 < t_2 < 1$. It should be obvious that the optimal strategies should satisfy $[t_1, t_2] \subset [0, 1-\gamma]$, and we shall assume this is the case from now on. Indeed, lowering $\lambda(t)$ when it is already 0 is useless.
Hence, the basic reproduction number of these control strategies is *constant* and is

$$R_0(\rho) = \frac{\bar{\lambda}_\rho}{\bar{\mu}} = \varphi_\rho(1) + 1 = R_0 - C\lambda_0\,. \quad (1.6)$$

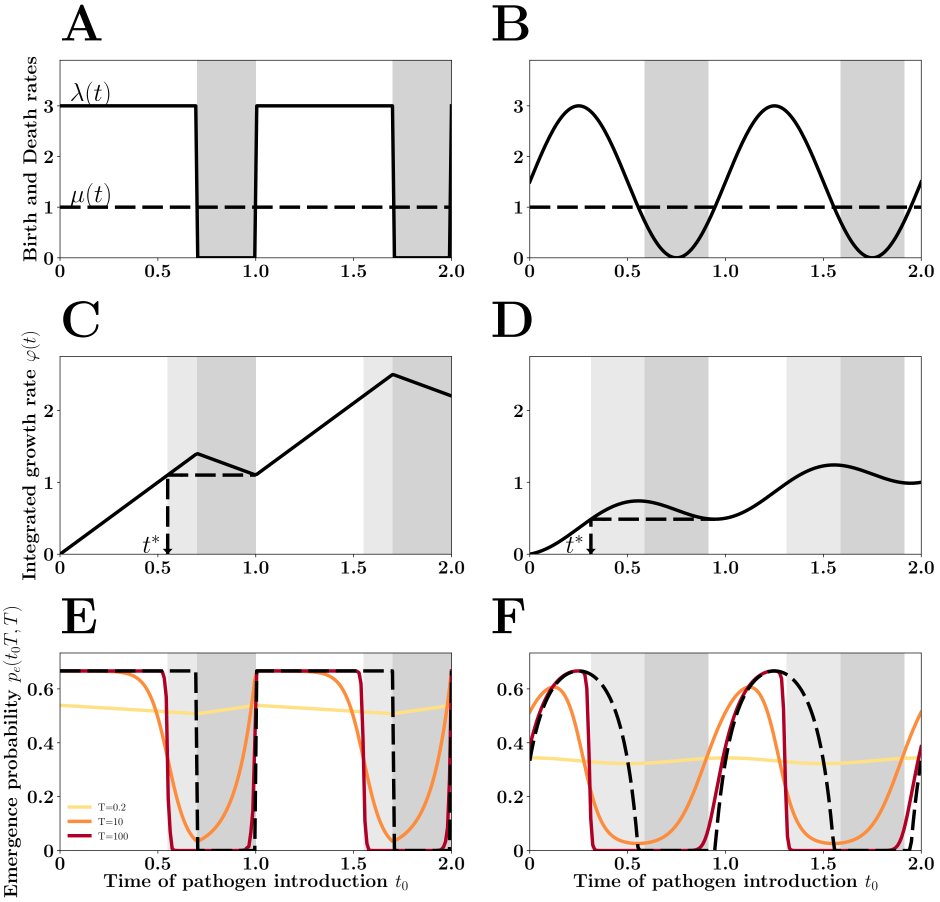

Figure A. The *winter is coming* effect. Pathogen *birth rate* $\lambda(t)$ (i.e. transmission rate) is assumed to vary periodically following a square wave (figures A, C and E) or a sinusoidal function (figures B, D and F). Pathogen *death rate* $\mu(t)$ (a function of recovery and death rates of the infected host) is assumed to be constant and equal to 1 in this figure. In the low transmission season we assume that transmission can become lower than the death rate ($\lambda(t) < \mu(t)$). The negative growth rate of the pathogen population during this period ($r(t) < 0$) creates a demographic trap (a drop in $\varphi(t)$, figures C and D) and reduces the probability of emergence at the end of the high transmision season (figures E and F). This *winter is coming* effect is indicated with a light gray shading between time t^* and the start of the low transmission season (figures C, D, E and F). This effect is particularly pronounced when the period of the fluctuations of the environment is large relative to the duration of the infection (i.e., when T is large, figures E and F).

Of course, if $C \geq \frac{R_0-1}{\lambda_0}$, then $R_0(\rho) \leq 1$, there is almost sure extinction for all these strategies and we are done. We shall therefore assume that $C < \frac{R_0-1}{\lambda_0}$ and this implies in particular that $C \leq 1-\gamma$.
Then, for a fixed large period T, one such strategy shall be close to optimal, since the extinction probabilities converge very fast.

Proposition 1.1. *Among the control strategies of type* (1.5) *with* $[t_1, t_2] \subset [0, 1-\gamma]$ *and fixed cost* $C = \rho_M(t_2 - t_1) < \frac{R_0-1}{\lambda_0}$, *there exists a continuum of optimal strategies that have all the minimal mean emergence probability*

$$\inf \langle p_{e,\infty,\rho} \rangle = \frac{R_0 - 1}{\lambda_0} - C\,. \tag{1.7}$$

The proof is straightforward, but tedious, and is done in section 6. In the course of the proof one can see that any strategy with cost C such that

$$t_1 \in \left[\frac{R_0 - 1 - C\lambda_0}{\lambda_0 - 1}, 1 - \gamma - C\right], \tag{1.8}$$

and $t_2 \leq 1-\gamma$ is optimal.
It is natural to compare these optimal strategies to the *naive strategy* that lowers λ on the whole interval $[0, 1-\gamma]$, that is $t_1 = 0$, $t_2 = 1-\gamma$:

$$\rho_n = \frac{C}{1-\gamma}\,\mathbf{1}_{(0<t<1-\gamma)}\,. \tag{1.9}$$

We have

$$\langle p_{e,\infty,\rho_n} \rangle = \left(\frac{R_0 - 1}{\lambda_0} - C\right)\frac{R_0}{R_0 - C\lambda_0}\,. \tag{1.10}$$

The comparison of the $\langle p_e \rangle$ obtained is done in figure B where we see the curves corresponding to (1.7) and (1.10). In contrast to the *naive strategy* the optimal control strategy allows to decrease the mean probability of emergence with higher investment in control.

1.2. **Sinusoidal wave.** We assume that death rate is constant $\mu(t) = 1$ and that the birth rate is sinusoidal

$$\lambda(t) = \lambda_0(1 + \sin(2\pi t))\,. \tag{1.11}$$

We can compute numerically the emergence probability $t_0 \to \langle p_e(t_0 T, T) \rangle$ and its mean. We observe that the *winter is coming* effect still applies, see figure A
The *control strategies* are of the type

$$\rho(t) = \rho_M\,\mathbf{1}_{(t_1<t<t_2)} \tag{1.12}$$

with $\rho_M \in [0,1]$ and $0 \leq t_1 \leq t_2 \leq 2$. We are going to compare control strategies with the same fixed cost $C > 0$. We have

$$C = \int_0^t \rho(s)ds = \rho_M(t_2 - t_1). \tag{1.13}$$

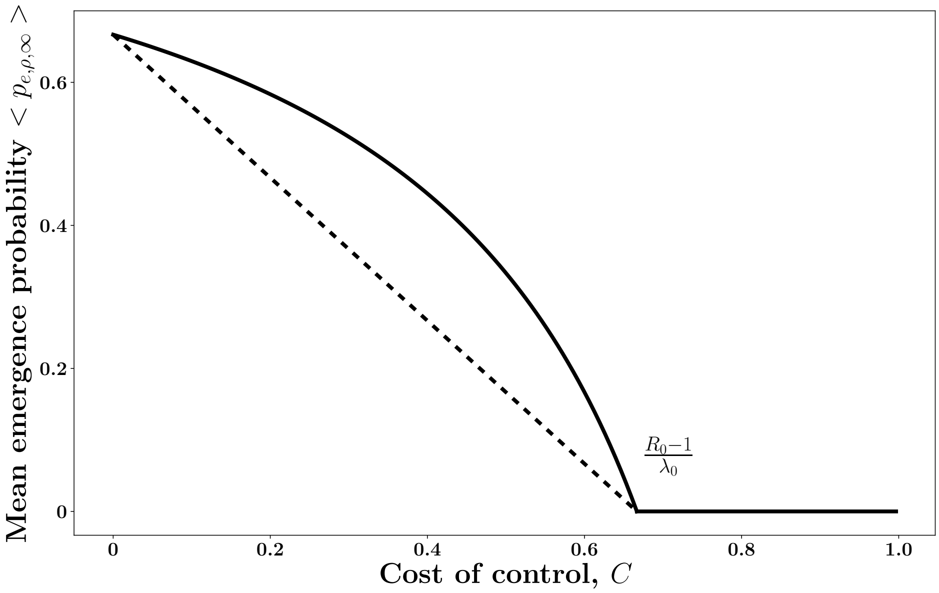

Figure B. The mean probability of emergence drops with higher investment in control. The efficacy of control varies wih the timing of the intervention. The *naive strategy* (in solid black, equation (1.10)) versus the *optimal strategy* (in dashed black, equation (1.7)) when the fluctuations follow a square wave (see also figure 4 in the main text). Parameters: $\lambda_0 = 3.0, \mu = 1, \gamma = 0.3, R_0 = 2.1$

Since $\Delta t = t_2 - t_1 \leq 1$, we shall assume that $C \leq \rho_M$. We decided to parameterize the control strategies by $(t_1, \rho_M) \in [0, 1] \times [C, 1]$.
In contrast with the square wave fluctuation scenario, the basic reproduction number $R_0(\rho)$ varies with the time at which control is applied during the high transmission season. Moreover, the optimal strategy, the one that has the lowest R_0 is *different* from the optimal strategy that has the lowest mean emergence (Figure 4 of main text).

1.3. **When the time of introduction is not uniformly distributed.** In the above sections we assumed that the time at which the initial infected host is introduced follows a uniform distribution. In other words we minimize:

$$< p_e >= \frac{1}{T} \int_0^T p_e(t_0 T, T)\, dt_0 \simeq \int_0^1 p_{e,\infty}(t_0)\, dt_0\,. \tag{1.14}$$

In this section we extend this approach and we allow the timing of introduction to follow a different distributiont $\delta(t)$. For instance we can assume that $\delta(t)$ is

proportional to the birth rate:

$$\delta(t) = \frac{1}{\lambda_0}\lambda(t) = 1 + \sin(2\pi t)\,. \tag{1.15}$$

In this scenario we have to minimize:

$$< p_e >= \frac{1}{T}\int_0^T p_e(t_0 T, T)\delta(t_0 T)\, dt_0 \simeq \int_0^1 p_{e,\infty}(t_0)\,\delta(t_0)\, dt_0\,. \tag{1.16}$$

Figure C illustrates the influence of the distribution of the timing of pathogen introduction on mean pathogen emergence. Note that the higher rate of introduction during the high transmission season increases dramatically the risk of pathogen emergence at that time. This explains why the timing of the optimal control is slightly delayed (closer to the maximal rate of pathogen transmission/introduction). In both cases the optimal strategy uses the *winter is coming effect* to reduce mean pathogen emergence.

2. Pathogen emergence for time varying multitype birth and death processes

Next we generalise the results obtained above in scenarios where the infected hosts may appear in different states (e.g. different host species, different states of the host). We consider a multitype birth and death processes where an individual i dies with rate μ_i and gives birth to an individual of type j at rate λ_{ij}. These rates are assumed to be non negative but time varying, so we obtain a time inhomogeneous Markov process on $\mathbb{N}^d$ with generator, for bounded functions f,

$$L_t f(x) = \sum_i x_i \left(\left(\sum_j \lambda_{ij}(t)(f(x+e_j) - f(x)) \right) + \mu_i(t)(f(x-e_i) - f(x)) \right) \tag{2.1}$$

with $x \in \mathbb{Z}^d$, and e_i the i-th base vector.

2.1. An ODE satisfied by the extinction probabilities.

Let $q_i(t_0, t) := \mathbb{P}\left(X_t = 0 \mid X_{t_0} = e_i\right)$ be the extinction probability at time t when the process starts with one individual of type i at time t_0. Since 0 is absorbing, we know that as $t \uparrow +\infty$, $q_i(t_0,t) \uparrow q_i(t_0,\infty) = \mathbb{P}\left(\exists t \geq t_0 : X_t = 0 \mid X_{t_0} = e_i\right)$, the *extinction probabilities*. The *emergence probabilities* are defined as

$$p_{e,i}(t_0) = 1 - q_i(t_0,\infty) = \mathbb{P}\left(\forall t \geq t_0, X_t > 0 \mid X_{t_0} = e_i\right). \tag{2.2}$$

Proposition 2.1. *The emergence probabilities are solutions of the system of ODE's*

$$p'_{e,i}(t_0) = \mu_i(t_0)p_{e,i}(t_0) - \sum_j \lambda_{ij}(t_0)(1 - p_{e,i}(t_0))p_{e,j}(t_0)\,. \tag{2.3}$$

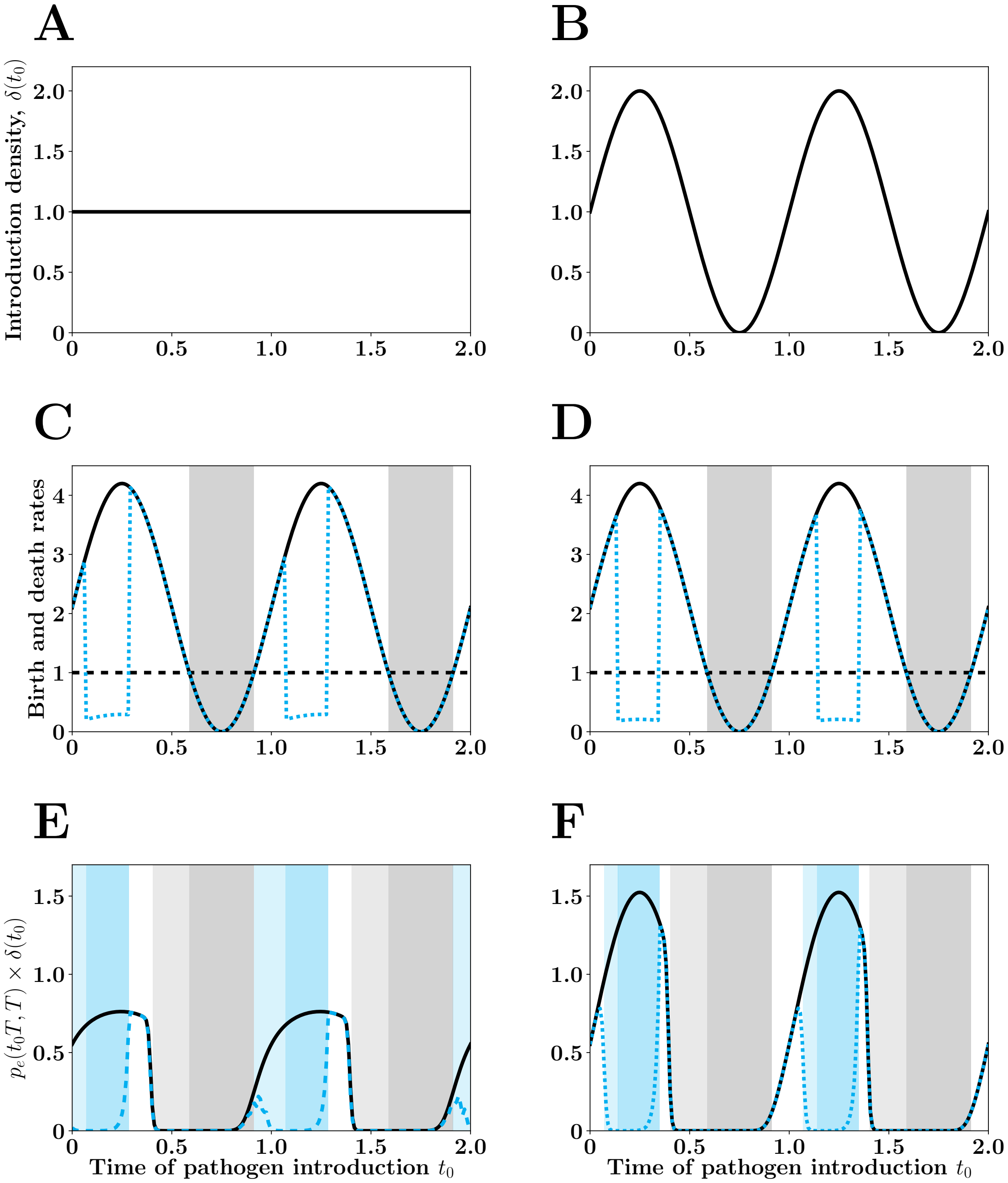

Figure C. Timing of introduction and pathogen emergence. We contrast two scenarios where the time of introduction follows a homogeneous distribution (A, C and E) or a sinusoid function (B, D and F) when transmission follows a sinusoid wave. In C and D we plot the sinusoidal fluctuations of pathogen transmission before (solid black line) and after (dashed blue line) the optimal control. In E and F we plot the probability of emergence before (solid black line) and after (dashed blue line) the optimal control. The blue shading indicates the timing at which optimal control is applied and the light blue shading refers to the *winter is coming* effect induced by the control. The function plotted in the last box is not the emergence probability but the product with the density $p_e(t_0T, T) * \delta(t_0)$ (our optimal control strategy minimizes the integral of this function).

Proof. Assume first that $t_0 = 0$. The first jump time, starting from e_i, has distribution

$$\mathbb{P}(T > t) = e^{-\Gamma_i(t)}\,, \quad \text{with} \quad \Gamma_i(t) := \int_0^t \left(\mu_i(s) + \sum_j \lambda_{ij}(s)\right) ds\,. \tag{2.4}$$

Therefore, conditioning by the value of T_i, we get

$$q_i(0,t) = \int_0^t \mathbb{P}(T \in ds, X_T \in dx)\mathbb{P}(X_t = 0 \mid X_s = x) \tag{2.5}$$

Thanks to the branching property,

$$\mathbb{P}(X_t = 0 \mid X_s = x) = \prod_i \mathbb{P}(X_t = 0 \mid X_s = e_i)^{x_i} = \prod_i q_i(s,t)^{x_i} \qquad (t \geq s). \tag{2.6}$$

We know that conditionally on $T = s$, we have one offspring of type j with probability $\frac{\lambda_{ij}(s)}{\mu_i(s)+\sum_k \lambda_{ik}(s)}$ and no offspring (i.e. death of type i) with probability $\frac{\mu_i(s)}{\mu_i(s)+\sum_k \lambda_{ik}(s)}$. Therefore,

$$q_i(0,t) = \int_0^t e^{-\Gamma_i(s)}(\mu_i(s) + \sum_j \lambda_{ij}(s)q_i(s,t)q_j(s,t))\,ds\,. \tag{2.7}$$

Since 0 is an absorbing set, we know that $q_i(t)$ increases to $q_i(\infty)$, and is non negative bounded since it is a probability. By dominated convergence, we can let $t \to +\infty$ in the above equality to get

$$q_i(0,\infty) = \int_0^\infty e^{-\Gamma_i(s)}\left(\mu_i(s) + \sum_j \lambda_{ij}(s)q_i(s,\infty)q_j(s,\infty)\right) ds\,. \tag{2.8}$$

In order to obtain $q_i(t_0,\infty)$ we replace in the preceding formula, $\lambda_{ij}(s)$ and $\mu_i(s)$ by $\lambda_{ij}(s+t_0)$ and $\mu_j(s+t_0)$.

$$q_i(t_0,\infty) = \int_0^\infty e^{-(\Gamma_i(s+t_0)-\Gamma_i(t_0))}\left(\mu_i(s+t_0) + \sum_j \lambda_{ij}(s+t_0)q_i(s+t_0,\infty)q_j(s+t_0,\infty)\right) ds\,, \tag{2.9}$$

$$= \int_{t_0}^{+\infty} e^{-(\Gamma_i(s)-\Gamma_i(t_0))}\left(\mu_i(s) + \sum_j \lambda_{ij}(s)q_i(s,\infty)q_j(s,\infty)\right) ds\,. \tag{2.10}$$

We can differentiate this equation to get

$$q_i'(t_0,\infty) = \left(\mu_i(t_0) + \sum_j \lambda_{ij}(t_0)\right) q_i(t_0,\infty) - \left(\mu_i(t_0) + \sum_j \lambda_{ij}(t_0)q_i(t_0,\infty)q_j(t_0,\infty)\right). \tag{2.11}$$

We easily check that the emergence probabilities $p_{e,i}(t_0) = 1 - q_i(t_0, \infty)$ satisfy (2.3).

□

Remark 2.2. *As usual the null function $p_{e,i}(t_0) = 0$ is always a solution of* (2.3) *and we can prove easily, by monotonicity arguments, that $p_{e,i}(t_0)$ is the largest solution, in $[0, 1]$ of equation* (2.3).
Let R_0 be the basic reproduction number for this birth and death process: we know, for example from [1] that $R_0 > 1$ iff $\forall i$, $\forall t_0$, $p_{e,i}(t_0) > 0$.
Eventually, observe that the ODE (2.3) *is a time varying Lotka-Volterra systems of equations, and we know that even in dimension* 2*, no exact solution is known.*

2.2. **A special case: when all rates are constant.** In this section we use the general expressions derived above in the special case where all the birth and death rates do not vary with time. We obtain the system:

$$p_{e,i} = \frac{\sum_j \lambda_{ij} p_{e,j}}{\mu_i + \sum_j \lambda_{ij} p_{e,j}} \tag{2.12}$$

We know that when $R_0 > 1$, the $p_{e,i}$ are positive solutions of this system.

In dimension $d = 1$. $R_0 > 1$ means $\lambda > \mu$ and $p_e = \frac{\lambda p_e}{\mu + \lambda p_e}$ yields $p_e = 1 - \mu/\lambda$.

In dimension $d = 2$. If $\lambda_{11} = \lambda_{22} = 0$ we get

$$p_{e,1} = \frac{\lambda_{12}\lambda_{21} - \mu_1\mu_2}{\lambda_{21}(\lambda_{12} + \mu_1)} \quad \text{with} \quad p_{e,2} = \frac{\lambda_{12}\lambda_{21} - \mu_1\mu_2}{\lambda_{12}(\lambda_{21} + \mu_2)} \tag{2.13}$$

and of course we have $R_0 > 1$ iff $\lambda_{12}\lambda_{21} - \mu_1\mu_2 > 0$.

The cyclic case. In a *cyclic* case an individual of type i can give rise to a single type j (noted $i + 1$), and no other type can give rise to $i + 1$. In this scenario we have:

$$\lambda_{i,i+1} > 0, \quad (1 \le i \le d - 1), \quad \lambda_{d,1} > 0 \tag{2.14}$$

and the other λ_{ij} are 0. To simplify notations we let $\lambda_{d,d+1} = \lambda_{d,1}$.
First observe that $R_0 > 1$ is equivalent to $\prod \lambda_{i,i+1} > \prod \mu_i$.
We have the formula,

$$p_{e,1} = \frac{\prod_{i=1}^{d} \lambda_{i,i+1} - \prod_{i=1}^{d} \mu_i}{\sum_{k=0}^{d-1} \prod_{i=1}^{k} \mu_i \prod_{i=k+1}^{d} \lambda_{i,i+1}} . \tag{2.15}$$

The other $p_{e,i}$ are given by a cyclic permutation of the preceding formula. Indeed, the equation (2.12) is now, with the convention that $p_{e,i} = p_{e,i\,mod(d)}$

$$p_{e,i} = \frac{\lambda_{i,i+1} p_{e,i+1}}{\mu_i + \lambda_{i,i+1} p_{e,i+1}} . \tag{2.16}$$

We start from $p_{e,1}$, we then plug in the formula for $p_{e,2}$, ... and when we come back to $p_{e,1}$ we simplify by $p_{e,1} > 0$ to get formula (2.15).

Effect of the type of the first infected host on p_e. We let $R_i = \lambda_{i,i+1}/\mu_i$ be a ratio that measure the relative transmission ability of host i.
In dimension $d = 2$ we get

$$p_{e,1} = \frac{R_1 R_2 - 1}{R_2(R_1 + 1)}, \quad p_{e,2} = \frac{R_1 R_2 - 1}{R_1(R_2 + 1)}. \tag{2.17}$$

Therefore

$$\frac{p_{e,1}}{p_{e,2}} = \frac{\varphi(R_1)}{\varphi(R_2)} \quad \text{with} \quad \varphi(R) = \frac{R}{R+1}. \tag{2.18}$$

Since φ is an increasing function $R_1 \geq R_2 \implies p_{e,1} \geq p_{e,2}$.

When $d > 2$, however, a higher value of R_i does not necessarily imply a higher value of $p_{e,i}$. For instance, in dimension $d = 3$ we get

$$p_{e,1} = \frac{R_1 R_2 R_3 - 1}{R_3(R_2(1 + R_1) + 1)}, \tag{2.19}$$

$$p_{e,2} = \frac{R_1 R_2 R_3 - 1}{R_1(R_3(1 + R_2) + 1)}, \tag{2.20}$$

$$p_{e,3} = \frac{R_1 R_2 R_3 - 1}{R_2(R_1(1 + R_3) + 1)}. \tag{2.21}$$

With $R_1 = 4 > R_3 = 2 > R_2 = 0.5$ we obtain $p_{e,3} = 0.4615 > p_{e,1} = 0.428 > p_{e,2} = 0.187$. The probability of emergence is lowest when the first infected host has a low R_i value (here host 2). But this example shows that the emergence probability does not rely only on the transmission ability of the first infected host because the host with the maximal value of R_i (here host 1) does not yield the highest value of $p_{e,i}$. The whole transmission cycle among the d types of hosts matters. Indeed, the pathogen is more likely to escape extinction if the *weak host* (here host 2) arrives later in the transmission cycle (2 steps after when host type 3 is introduced first, compared to one step after when host type 1 is introduced first). This effect is akin to the *winter is coming* effect we discussed above. Indeed, this *weak host is coming* effect is due to the alternation between good and bad hosts during the transmission cycle.

2.3. **The numerical approach of Bacaër and Ait Dads.** In principle, there should be no problem to numerically approximate $p_{e,i}$ a solution of (2.3). However, we do not know how to fix boundary counditions. If we knew a way to fix them, that would mean that the values of $p_{e,i}(0)$ are already known.
[2] established an approximation of the extinction probabilities $p_{e,i}$ by combining the method of characteristics and Kolmogorov's formard equation. We used these to perform our computer simulations. The method is the following.

Let τ be a large number, at least large with respect to the period T. Let $Y^{(\tau)}$ be the solution, with initial condition $Y_i^{(\tau)}(0) = 1$, of

$$\frac{d}{ds} Y_i^{(\tau)}(s) = -\mu_i(\tau - s) Y_i^{(\tau)}(s) + \sum_j \lambda_{ij}(\tau - s)(1 - Y_i^{(\tau)}(s)) Y_j^{(\tau)}(s)\,. \qquad (2.22)$$

Then, when τ is large enough, $Y_i^{(\tau)}(\tau - t_0)$ is close to $p_{e,i}(t_0)$ as illustrated in figure D : if you *reverse time* you look at the top curve from right to left that is you look at $t_0 \to Y_i^{(\tau)}(\tau - t_0)$ (and then you get a very close approximation to $t_0 \to p_{e,i}(t_0)$).

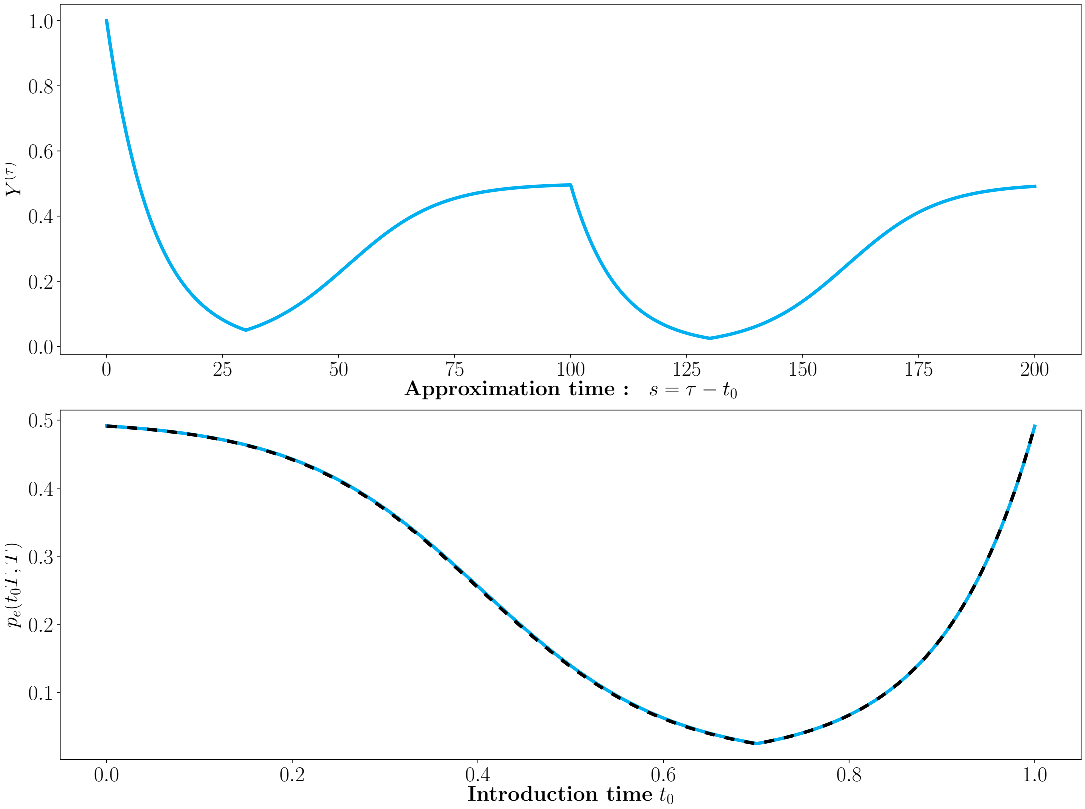

Figure D. Numerical calculation of $p_e(t_0T, T)$. The top graphic is $Y^\tau(s)$ for a one dimensional birth and death process with birth rate $\lambda(s) = 0.2\,\mathbf{1}_{(0<s<0.7)}$, $\mu(s) = 0.1$, the period $T = 100$ and $\tau = 2T$. We see that it stabilizes very fast to the periodic solution. The bottom graphic shows that the emergence probability $p_e(t_0T, T)$ as a function of t_0, in dotted black, coincides numerically with its approximation $Y^\tau(\tau - t_0)$, in solid blue, that is the top curve taken right to left on one period.

We are now going to give a probabilistic proof of the approximation

$$Y_i^{(\tau)}(\tau - t_0) \simeq p_{e,i}(t_0)\,. \tag{2.23}$$

One interesting byproduct of this proof, that makes it a complement to the proof of [2], is that it gives convergence rates.
Indeed, let us look again at extinction probabilities. As in the proof of Proposition 2.1, for $0 \leq t_0 \leq \tau$ we have

$$q_i(t_0, \tau) = \mathbb{P}\left(X_\tau = 0 \mid X_{t_0} = e_i\right) \tag{2.24}$$

$$= \int_{t_0}^{\tau} e^{-(\Gamma_i(s)-\Gamma_i(t_0))} \left(\mu_i(s) + \sum_j \lambda_{ij}(s) q_i(s,\tau) q_j(s,\tau)\right) ds\,. \tag{2.25}$$

Differentiating with respect to t_0, we get that $t_0 \to q_i(t_0, \tau)$ satisfy the same differential equations (2.11) as $q_i(t_0, \infty)$, but with the boundary conditions $q_i(\tau, \tau) = 0$. In terms of emergence probabilities that means that $Z_i^{(\tau)}(t_0) = 1 - q_i(t_0, \tau)$ satisfy the differential equations (2.3) with the boundary condition $Z_i^{(\tau)}(\tau) = 1$. By uniqueness of ODE solutions with locally Lipschitz coefficients, this implies that

$$Y_i^{(\tau)}(\tau - t_0) = Z_i^{(\tau)}(t_0) \qquad (0 \leq t_0 \leq \tau)\,. \tag{2.26}$$

We have already observed that since 0 is an absorbing point for the branching process, $q_i(t_0, \infty) = \lim_{\tau \to +\infty} q_i(t_0, \tau)$. And this translates immediately to

$$p_{e,i}(t_0) = \lim_{\tau \to +\infty} Z_i^{(\tau)}(t_0) = \lim_{\tau \to +\infty} Y_i^{(\tau)}(\tau - t_0)\,. \tag{2.27}$$

We can even show that this convergence happens exponentially fast. Indeed, let

$$\kappa = \inf_i \inf_s (\mu_i(s) + \sum_j \lambda_{ij}(s)) > 0\,. \tag{2.28}$$

Then, remembering that $0 \leq q(s, \tau) \leq 1$, we have

$$\left|p_{e,i}(t_0) - Y_i^{(\tau)}(\tau - t_0)\right| = \int_{\tau}^{\infty} e^{-(\Gamma_i(s)-\Gamma_i(t_0))} \left(\mu_i(s) + \sum_j \lambda_{ij}(s) q_i(s,\tau) q_j(s,\tau)\right) ds \tag{2.29}$$

$$\leq \int_{\tau}^{\infty} e^{-(\Gamma_i(s)-\Gamma_i(t_0))} \left(\mu_i(s) + \sum_j \lambda_{ij}(s)\right) ds \tag{2.30}$$

$$= \int_{\tau}^{\infty} e^{-(\Gamma_i(s)-\Gamma_i(t_0))} \Gamma_i'(s)\, ds = e^{\Gamma_i(t_0)} \left[-e^{-\Gamma_i(s)}\right]_{\tau}^{\infty} \tag{2.31}$$

$$= e^{-(\Gamma_i(\tau)-\Gamma_i(t_0))}\,. \tag{2.32}$$

Since we have the lower bound

$$\Gamma_i(\tau) - \Gamma_i(t_0) = \int_{t_0}^{\tau} \left(\mu_i(s) + \sum_j \lambda_{ij}(s) \right) ds \geq \kappa(\tau - t_0)\,, \tag{2.33}$$

we obtain

$$\sup_{t_0 \in [0,T]} \left| p_{e,i}(t_0) - Y_i^{(\tau)}(\tau - t_0) \right| \leq e^{-\kappa(\tau - T)}\,. \tag{2.34}$$

If we consider the example of figure D, we can take $\kappa = 0.1$, we have $T = 100$ and we want to take $\tau = nT$, a finite number of periods, large enough to ensure a precision of at least 10^{-3}. It is enough to have $\kappa(\tau - T) > 3\log(10)$ and thus $n - 1 \geq \frac{3\log(10)}{\kappa T} = 0.69$. Therefore, having the approximation $Y^{(\tau)}$ run for $\tau = 2T$ (2 periods) is largely enough.

2.4. **Asymptotic results for large periods:** $T \to +\infty$**.** When we rescaled functions $\lambda_{ij}(t) := \lambda_{ij,T}(tT)$ and $\mu_i(t) := \mu_{i,T}(tT)$ we observe the same phenomenom as in the one dimensional scenario. The emergence probabilities $p_{e,i}(t_0T, T)$ are very close to 0 on sub intervals. Besides, when they are not 0 they are very close to the *guess* $\pi_{e,i}(t_0)$ which are obtained by substituting in the formulas giving the emergence probabilities for constant rates, the rates by their values at time t_0, the time of introduction of the infected individual of type i (see figure 1 of main text). For example, in dimension $d = 2$, with$\lambda_{11} = \lambda_{22} = 0$, we have

$$\pi_{e,1}(t_0) := \frac{\lambda_{12}(t_0)\lambda_{21}(t_0) - \mu_1(t_0)\mu_2(t_0)}{\lambda_{21}(t_0)(\lambda_{12}(t_0) + \mu_1(t_0))} \tag{2.35}$$

The $\pi_{e,i}(t_0)$ are solutions of (2.12), where the rates λ_{ij}, μ_i are replaced by their values at t_0.
Unfortunately, we are unable to determine a set A such that

$$\lim_{T \to +\infty} p_{e,i}(t_0T, T) = \pi_{e,i}(t_0)\, \mathbf{1}_{(t_0 \in A)}\,. \tag{2.36}$$

Indeed, we are unable to prove that the sequence of functions $T \to (p_{e,i}(.T, T))$ is compact in the set of continous functions from $[0, 1]$ to $\mathbb{R}$ by applying Arzela-Ascoli's theorem : the functions $p_{e,i}(.T, T)$ are uniformly bounded together with the functions functions $\lambda_{i,j}$, μ_i are bounded (periodic and locally bounded), but the derivatives have a T factor that prevents them to be bounded. This is exactly what happens in dimension 1 where we proved that the limit was discontinuous.
However, let us consider the limit of a subsequence, i.e. assume that for a sequence $T_n \to +\infty$, the functions $p_{e,i}(t_0T_n, T_n)$ converge almost everywhere on $[0, 1]$ to the functions $\alpha_i(t_0)$.

Proposition 2.3. *For every $t_0 \in [0, 1]$ such that the α_i are right continuous at t_0, either $\alpha_i(t_0) = \pi_{e,i}(t_0)$, either $\alpha_i(t_0) = 0$. In particular, whenever $\pi_{e,i}(t_0) = 0$, we have $\alpha_i(t_0) = 0$.*

Proof. We are going to prove that $\alpha_i(0)$ satisfy the same equations as the guess $\pi_{e,i}(0)$. The general result can then be deduced by considering the shifted rates $\lambda_{ij}(.) = \lambda_{ij}(. + t_0)$.
Let us rewrite equation (2.9), taking into account periodicity, of all the functions $\lambda_{ij}, \mu_i, y_{i,T}$ and so $\Gamma_{i,T}(s + kT) = k\Gamma_{i,T}(T) + \Gamma_{i,T}(s)$,

$$\begin{aligned}1 - p_{e,i}(0,T) &= \int_0^{+\infty} e^{-\Gamma_{i,T}(s)} \left(\mu_{i,T}(s) + \sum_j \lambda_{ij,T}(s)(1 - p_{e,i}(s,T))(1 - p_{e,j}(s,T)) \right) ds\,, \\ &= \sum_{k=0}^{+\infty} \int_{kT}^{(k+1)T} \cdots \\ &= \sum_{k=0}^{+\infty} e^{-k\Gamma_{i,T}(T)} \int_0^T \cdots \\ &= \frac{1}{1 - e^{-\Gamma_{i,T}(T)}} \int_0^T e^{-\Gamma_{i,T}(s)} \left(\mu_{i,T}(s) + \sum_j \lambda_{ij,T}(s)(1 - p_{e,i}(s,T))(1 - p_{e,j}(s,T)) \right) ds\,.\end{aligned}$$

With the change of variables $t = s/T$, we get

$$1-p_{e,i}(0,T) = \frac{T}{1 - e^{-T\Gamma_i(1)}} \int_0^1 e^{-T\Gamma_i(t)} \left(\mu_i(t) + \sum_j \lambda_{ij}(t)(1 - p_{e,i}(tT,T))(1 - p_{e,j}(tT,T)) \right) dt \tag{2.37}$$

Letting $T = T_n$, we can take limits, and we recognise a Laplace integral

$$\begin{aligned}1 - \alpha_i(0) &= \lim_{n\to+\infty} T_n \int_0^1 e^{-T_n\Gamma_i(t)} \left(\mu_i(t) + \sum_j \lambda_{ij}(t)(1 - \alpha_i(t))(1 - \alpha_j(t)) \right) dt \\ &= \frac{1}{\Gamma_i'(0)} \left(\mu_i(0) + \sum_j \lambda_{ij}(0)(1 - \alpha_i(0))(1 - \alpha_j(0)) \right), \\ &= \frac{\mu_i(0) + \sum_j \lambda_{ij}(0)(1 - \alpha_i(0))(1 - \alpha_j(0))}{\mu_i(0) + \sum_j \lambda_{ij}(0)}\,.\end{aligned}$$

These are exactly the equations satisfied by $\pi_{e,i}(0)$. □

2.5. **A direct proof of Kendall's formula for the emergence probability.** We know that $p_e(t_0)$ is a solution of the ODE (2.3) :

$$p_e' = p_e(\mu - \lambda(1 - p_e))\,. \tag{2.38}$$

We can solve this equation by letting $v(t_0) = \frac{1}{p_e(t_0)}$ that satisfies

$$v' = (\lambda - \mu)v - \lambda\,. \tag{2.39}$$

Therefore for a constant C:

$$v(t_0) = e^{\varphi(t)}\left(C - \int_0^{t_0} \lambda(s)e^{-\varphi(s)}\, ds\right). \tag{2.40}$$

Since $0 \leq p_e(t_0) \leq 1$ we get that $C = \int_0^{\infty} \lambda(s)e^{-\varphi(s)}\, ds$ and therefore, with an integration by parts,

$$\frac{1}{p_e(t_0)} = e^{\varphi(t_0)} \int_{t_0}^{\infty} \lambda(s)e^{-\varphi(s)}\, ds = 1 + e^{\varphi(t_0)} \int_{t_0}^{\infty} \mu(s)e^{-\varphi(s)}\, ds\,, \tag{2.41}$$

which is exactly equation (4) of the main text (Kendall's formula).

3. **A vector borne disease : Zika virus**

We want to determine the probability of emergence of Zika Virus, a newly emerging vector borne disease of humans. Our starting point is the epidemioloical model of Zika used in previous studies [3, 4, 5]. The epidemiological dynamics in the human population is described by an SEIR model : susceptible individuals S^H, exposed indivuals E^H, infected individuals I^H and recovered/removed individuals R^H. The epidemiological dynamics in the vector population is described by and SEI model with compartments S^V, E^V, I^V. This yields the following deterministic dynamics:

$$\frac{dE^H}{dt} = \lambda_{I^V,E^H} I^V - \mu_{E^H} E^H\,, \qquad \frac{dE^V}{dt} = \lambda_{I^H,E^V} I^H - \mu_{E^V} E^V \tag{3.1}$$

$$\frac{dI^H}{dt} = \lambda_{E^H,I^H} E^H - \mu_{I^H} I^H\,, \qquad \frac{dI^V}{dt} = \lambda_{E^V,I^V} E^V - \mu_{I^V} I^V \tag{3.2}$$

The probability of a major outbreak is the probability of emergence in a birth death process with 4 types. By labelling the states E^H, I^H, E^V, I^V as $1, 2, 3, 4$ we can write this system as:

$$\frac{dx_i}{dt} = -\mu_i x_i + \sum_j \lambda_{i,j} x_j\,, \tag{3.3}$$

and the generator of the stochastic process is (2.1). Note that we are in a *cyclic* case : $\lambda_{i,j} = 0$ only if $j = i+1\, mod(d)$. The death rates μ_i are functions of mortality and recovery rates. For instance, $\mu_{E^H} = \gamma^H + \mu^H$ where $\frac{1}{\gamma^H}$ is the expected incubation time of the infection in humans, usually between 5 and 9 days, and $\frac{1}{\mu^H}$ is the expected lifespan of humans, generally taken to be 75 years. The total number of humans $N^H = S^H + E^H + I^H + R^H$ is assumed to be constant over time. Crucially, the seasonality, the periodicity in time, of some parameters comes only through their dependance on temperature. More precisely, a parameter $\mu_i, \lambda_{i,j}$ is periodic iff it is a function of temperature T, which is periodic (with period one year). The parameter values infered from observed epidemiological dynamics agree very well

among the three studies we used [3, 4, 5]. All the parameter values used in our models are summed up in Table A.
The seasonality comes through temperature only, which follows a sinusoid:

$$T^{\circ}(t) = T^{\circ}_{moy} + \frac{A_{T^{\circ}}}{2}\sin\left(\frac{2\pi}{T}\,t\right), \tag{3.4}$$

with T°_{moy} the mean temperature and $A_{T^{\circ}}$ the amplitude of the variation in temperature.
The number of vectors per human $M(T^{\circ}) = \frac{N^V}{N^H}$ is assumed to reach a maximum M_{max} at the optimal temperature T°_{opt}. Following [5] we assume the number of vectors per human drops away from the optimal temperature:

$$M(T^{\circ}) = M_{max}\exp\left(-\frac{(T^{\circ} - T^{\circ}_{opt})^2}{2\sigma^2_{T^{\circ}}}\right) \tag{3.5}$$

with the optimal temperature for vector reproduction and $\sigma^2_{T^{\circ}} \in [10, 50]$ a spread factor (the "variance" for this gaussian shape).
In this model the *winter* (i.e. the low transmission season) is defined to be the set of times t_0 for which the computation of R_0 would yield $R_0 < 1$ if the parameters were to be frozen at their values $\lambda_{ij}(t_0)$ and $\mu_i(t_0)$. In other words it is the set of time for which the product of the birth rates is less than the product of the death rates (see equation (25)).

$$W = \left\{t_0 : \prod \lambda_{ij}(t_0) < \prod \mu_i(t_0)\right\}. \tag{3.6}$$

Unfortunately, unlike the model in dimension 1, where it is defined as the complementary of the set A of Proposition 6.1, the timing of the *winter is coming* effect cannot be identified easily (see Figure A and equation (27)). We decided to define the *winter is coming* time zone to be the set of times for which the emergence probability is less than a fixed threshold $\delta = 0.05$

$$WIC = \{t_0 : p_e(t_0T, T) < \delta = 0.05\}. \tag{3.7}$$

Model I. In this simple model, as in [4], there is a single rate that varies with time. We assume that seasonality affects only the population size of vectors and, consequently, the only rate that varies with time is $\lambda_{I^H,E^V} = \beta\frac{N_V}{N_H}$. In the figure 5 of the main text we show the influence of a small shift in the mean temperature on the probability of pathogen emergence after the introduction of a single infected human in the *exposed* state. This figure illustrates that we recover the winter is coming effect when the amplitude of the seasonal variation are large enough.
In the figure E we illustrate the influence of the state of the introduced pathogen: E^H, I^H, E^V or I^V. This figure shows that the probability of emergence is much higher when the pathogen is introduced in a human host (black lines) rather than in a vector (red lines) because the duration of infection is higher in humans. Besides, the probability of emergence is higher if the introduced host is already infectious

	**Definition**	**Model I**	**Model II**
λ_{E^H,I^H}	Rate of transition between E^H and I^H	0.143	
λ_{I^H,E^V}	Rate of transition between I^H and E^V	$bM(T^\circ)$	$bM(T^\circ)\,\beta(T^\circ)$
λ_{E^V,I^V}	Rate of transition between E^V and I^V	0.125	
λ_{I^V,E^H}	Rate of transition between I^V and E^H	b	$b\,\beta(T^\circ)$
b	Biting rate of the vector	0.32	
μ_{E^H}	Recovery rate + mortality rate of E^H	0.143	
μ_{I^H}	Recovery rate + mortality rate of I^H	0.2	
μ_{E^V}	Mortality rate of E^V	0.525	
μ_{I^V}	Mortality rate of I^V	0.4	
T	Period of the fluctuation	365 days	
T°_{moy}	Mean temperature	27° and 29°	25°
T°_{opt}	Optimal temperature for the reproduction of the vector	32°	
A_{T°	Amplitude of the variation in temperature	4°	8°
M_{max}	Maximal value of the number of vectors per human $\frac{N^V}{N^H}$	10	
$\sigma^2_{T^\circ}$	Variance for the distribution of the number of vectors per human around the optimal temperature T°_{opt}	14.05	

Table A. Definition and values of the parameters used in the Zika models. All the rates (λ, μ and b) are in $(days)^{-1}$.

(dashed line) rather than exposed (full line) because the pathogen can readily be transmitted from an infectious host and does not need to wait the end of the incubation time.

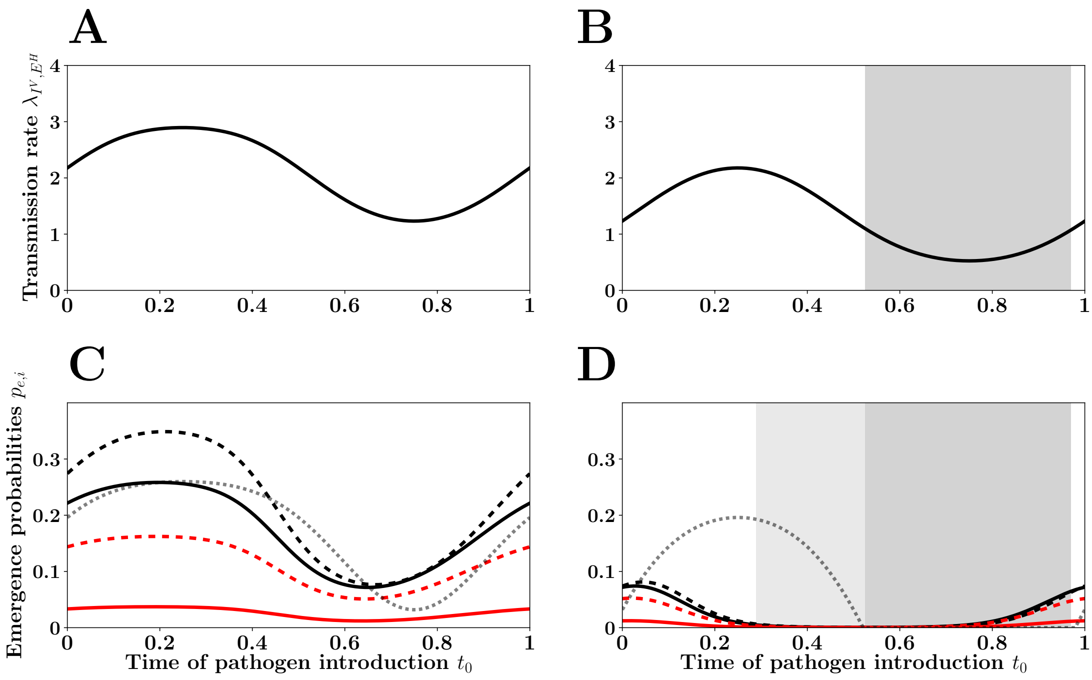

Figure E. Effect of the state of the introduced pathogen on the probability of Zika emergence (model I). The top figures (A and B) show the seasonal variations in λ_{I^V,E^H}, the transmission rate from humans to the vectors because of the fluctuations the density of vectors in two habitats (this illustrates the effect of *space* on Zika emergence): a minor variation in mean temperature, 29 °C (A and C) versus 27 °C (B and D), has a massive impact on transmission and, consequently, on pathogen emergence. In C and D we illustrate the effect of the *time* of introduction t_0 on Zika emergence and of the *state* in which it is introduced : in solid black an exposed human (E^H), in dashed black an infected human (I^H), in solid red an exposed mosquito (E^V), in dashed red an infected mosquito (I^V).

Model II. In this second model we allow more transition rates to depend on the temperature as in [5] and [3]. We chose to have two transmission parameters depending on the temperature λ_{I^H,E^V} and λ_{I^V,E^H}. They both have a common factor, the *biting rate*, modeled after [6] as:

$$\beta(T^\circ) = 0.1444\, T^\circ(T^\circ - 12.286)\sqrt{32.461 - T^\circ}\,. \tag{3.8}$$

This yields:

$$\lambda_{I^H,E^V}(T^\circ) = \tilde{\beta} M(T^\circ)\, \beta(T^\circ) \qquad \lambda_{I^V,E^H}(T^\circ) = b\, \beta(T^\circ)\,. \tag{3.9}$$

Figure F shows the fluctuations in both λ_{I^H,E^V} and λ_{I^V,E^H} throughout the season. The function $\beta(T^\circ)$ induces major drops in transmission when the temperature is too low but also when it is too high relative to T°_{opt}. These fluctuations induce two peaks in the probability of pathogene emergence. The optimal timing of the control can have a massive impact on the risk of pathogen emergence during the period of control but also before. This confirms the relevance of the *winter is coming* effect in a realistic model of Zika emergence.

4. **Pulse interventions**

4.1. **A pulse vaccination model.** We want to determine the probability of pathogen emergence in a seasonal environment when vaccination is applied periodically (every year) in a pulse occuring at time δ during the year. The question is : *When is the best time δ to vaccinate?* Following [7] we use a one dimensional linear birth and death process with a constant death rate $\mu(t) = 1$ and a time varying birth rate:

$$\lambda(t) = \beta(t) Q(t). \tag{4.1}$$

The function $\beta(t)$ refers to the seasonal transmission rate:

$$\beta(t) = \beta(1 + \sigma \cos(2\pi t))\,, \tag{4.2}$$

The function $Q(t)$ is the proportion of unvaccinated in a deterministic model of population with death rate and birth rate equal to μ, with $Q(t) + V(t) = 1$ and thus for $t \in [0, 1)$:

$$\frac{dQ}{dt} = \mu - \mu Q \tag{4.3}$$

$$\frac{dV}{dt} = -\mu V\,. \tag{4.4}$$

The solution of this ODE is:

$$Q(t) = Q(0)e^{-\mu t} + 1 - e^{-\mu t}\,, \tag{4.5}$$

Let us assume that a pulse vaccination is applied at the end of the season (at time 1). The number of unvaccinated is instantly decreased by a factor $q \in (0, 1)$:

$$Q(1) = qQ(1-) = q(Q(0)e^{-\mu} + 1 - e^{-\mu})\,. \tag{4.6}$$

Then we make the same operation on intervals $[1, 2[, [2, 3[, \ldots$. It is easy to see that the function constructed this way is asymptotically close to the 1-periodic function:

$$Q(t) = 1 - e^{-\mu t} \frac{1 - q}{1 - qe^{-\mu}} \qquad (t \in [0, 1))\,, \tag{4.7}$$

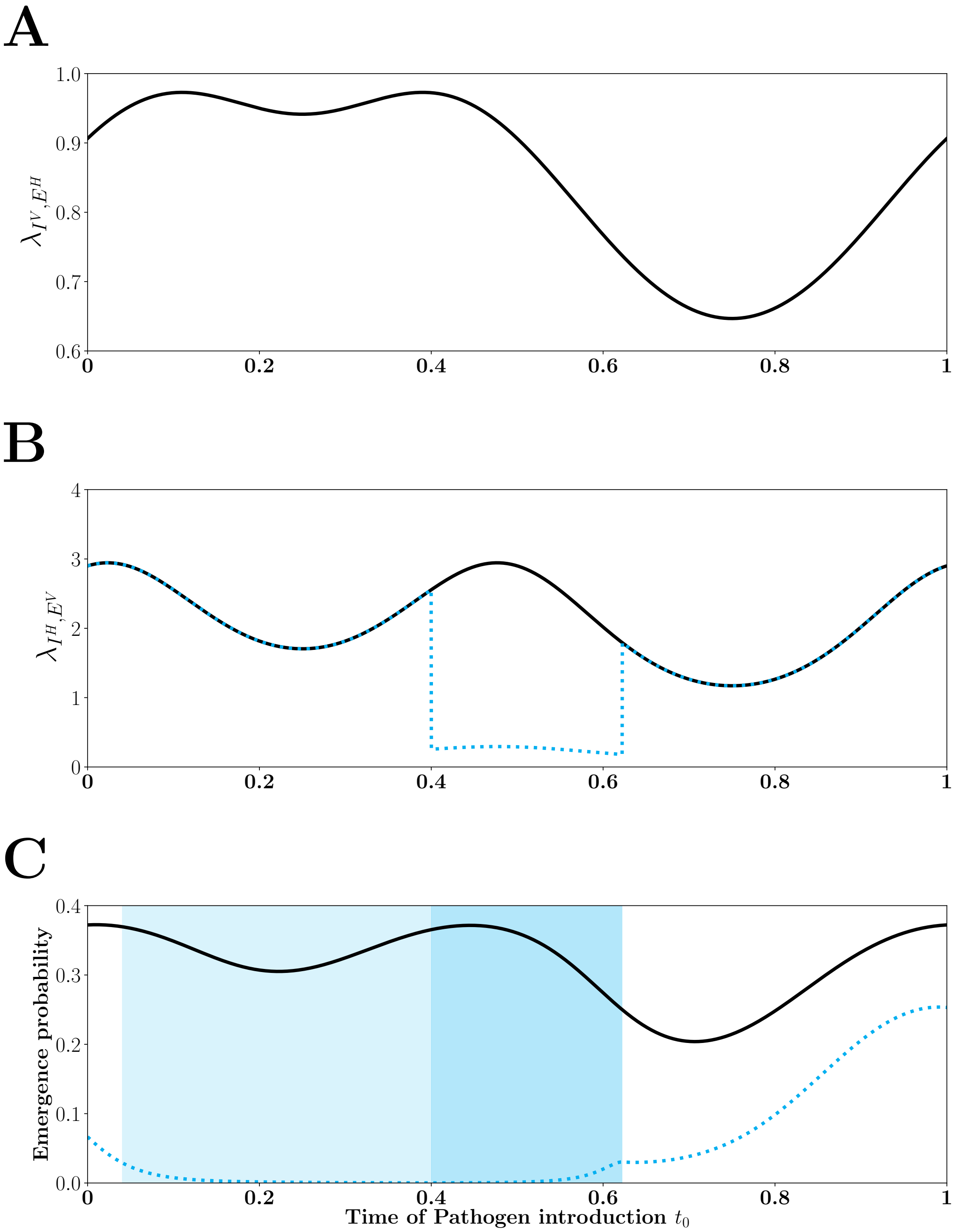

Figure F. Probability of Zika emergence (model II). In model II both the density of mosquito vectors (equation 3.5)and the biting rate (equations 3.8 and 3.9) fluctuate throughout the season. This yields a fluctuation in λ_{I^V,E^H} (A) and λ_{I^H,E^V} (B). Without control the probability of Zika emergence (in solid black) is always above 0.2 because transmission remains relatively high in the low-transmission season (see also figure 5A and 5C). The optimal control operates only on the density of mosquitoes, the blue dotted curve in B, during the time interval $[t_1, t_2] = [0.4, 0.62]$ (blue shading in C) and results in an important *winter is coming* effect (light blue shading in C).

which is the function (4.4) of [7].

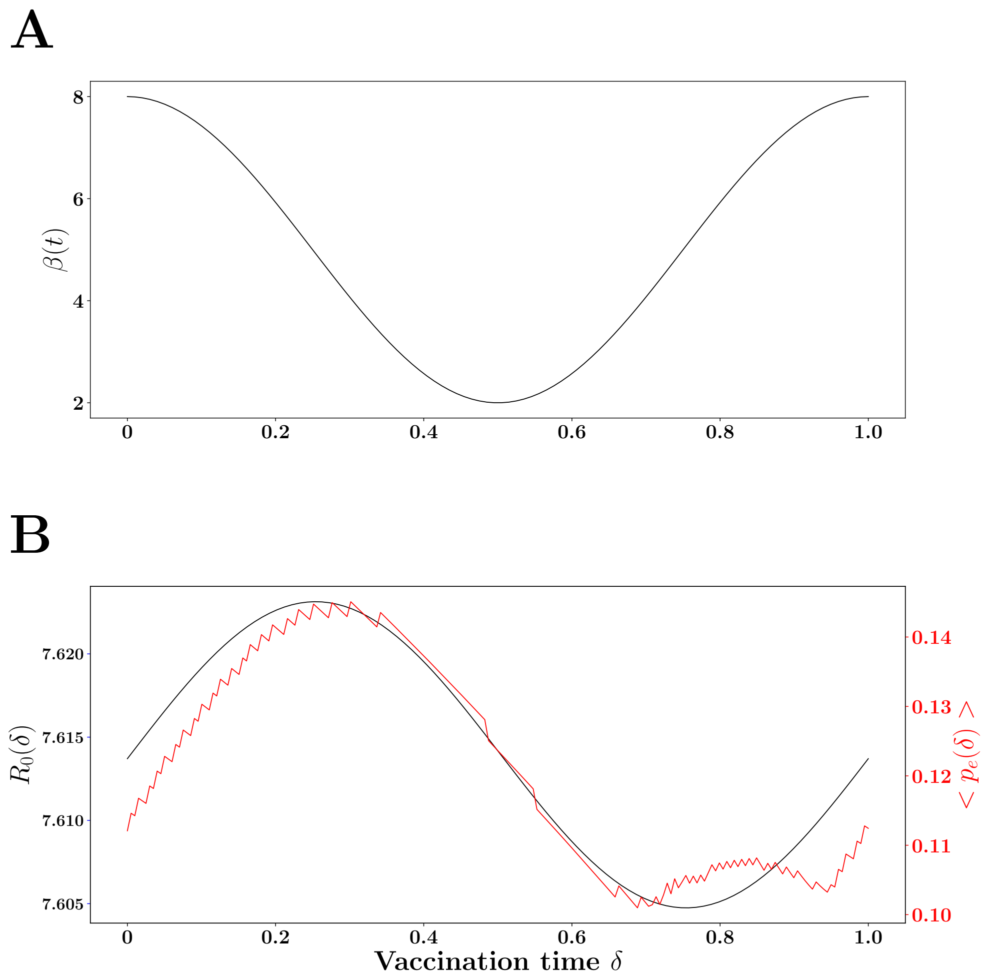

Figure G. Effect of the timing of the pulse vaccination on R_0 and the mean probability of pathogen emergence. The upper curve is $\beta(t)$, pathogen transmission rate before vaccination (figure A): it enables to identify the high and low transmission seasons. The mean emergence probability without pulse vaccination is $\langle p_e \rangle = 0.75$. In figure B we plot $R_0(\delta)$, the basic reproduction number (black line), and $< p_e(\delta) >$, the mean extinction probability (red line) against δ, the timing of the pulse vaccination (i.e. the amount of time after the start of the high transmission period). Parameters: $\sigma = 0.6$, $\beta = 5.0$, $q = 0.6$, $\mu = 0.16$.

Next we assume that the pulse vaccination may be applied at another point in time during the year. The parameter $\delta \in (0,1)$ refers to the shift between the seasonal fluctuation and the vaccination pulse. We consider $Q_\delta(t) = Q(t-\delta)$ and $\lambda_\delta(t) = Q_\delta(t)\beta(t)$. We obtain

$$R_0(\delta) = 1 - a\frac{1-e^{-\mu}}{\mu} - a\frac{\sigma(1-e^{-\mu})}{2\pi(1+\bar{\mu}^2)}(\bar{\mu}\cos(2\pi\delta) - \sin(2\pi\delta)) \tag{4.8}$$

with $\bar{\mu} = \frac{\mu}{2\pi}$ and $a = \frac{1-q}{1-qe^{-\mu}}$.

The optimal shift,that minimizes $R_0(\delta)$, has been computed by [7] and is $\delta_o = \frac{3}{4} + \frac{1}{2\pi}\arctan(\bar{\mu}) \simeq 0.75$ for small μ (say $\mu = 0.16$). This result is in accordance with [7]. They found by doing some numerical stochastic simulations that $\delta = 0.75$ was a better choice than $\delta = 0.5$ to decrease the mean emergence probability $< p_e >$. Our calculation, however, allows to compute precisely the optimal δ using the original formula of [8] adapted to seasonal environment, equation (4). We find that the optimal delta δ_*, that minimizes the mean emergence probability $\langle p_e \rangle$, for the set of parameters given by [7], $\delta_* = 0.69$, see figure G. Again, we see that the control strategy that minimizes $\langle p_e \rangle$ is not the same as the strategy that minimizes R_0.

4.2. **A pulse treatment model.** We want to determine the probability of pathogen emergence in a seasonal environment when an antibiotic treatment is applied periodically (every year) in a pulse occuring at time δ during the year. The question is : *When is the best time δ to treat?*

In the absence of treatment we use the same one dimensional model where the death rate $\mu(t)$ is constant and the birth rate is:

$$\lambda(t) = \beta(1 + \cos(2\pi t)) \,. \tag{4.9}$$

The treatment pulse occurs at time $\delta \in (0,1)$: there is an increase of $\mu(t)$ from 1 to $1 + \Delta\mu$ during the time interval $(\delta, \delta + \Delta t)$. If Δt is small enough, this is close to a dirac pulse, and the effect is to reduce quasi instantly the number of infected by the factor $c = e^{-\Delta t \Delta\mu}$. Since $\mu_\delta(t) = 1 + \Delta\mu\,\mathbf{1}_{(\delta<t<t+\Delta t)}$, the basic reproduction number is given by:

$$R_0(\delta) = \frac{\bar{\lambda}}{\bar{\mu_\delta}} = \frac{\beta}{1+\Delta t\Delta\mu} \,. \tag{4.10}$$

Observe that $R_0(\delta)$ *does depend* on the pulse intensity $\Delta t\Delta\mu$, but *does not depend* on the timing δ of the pulse.

Nevertheless, the emergence probability *does depend* on δ, and we can determine the optimal δ_* that minimizes the mean emergence probability $< p_e(\delta) >$ see Figure H. This scenario illustrates again that minimizing $R_0(\delta)$ or minimizing $< p_e(\delta) >$ does not yield the same optimal control strategy. We also recover that the optimal timing of the control is just before the high transmission season (compare G and H).

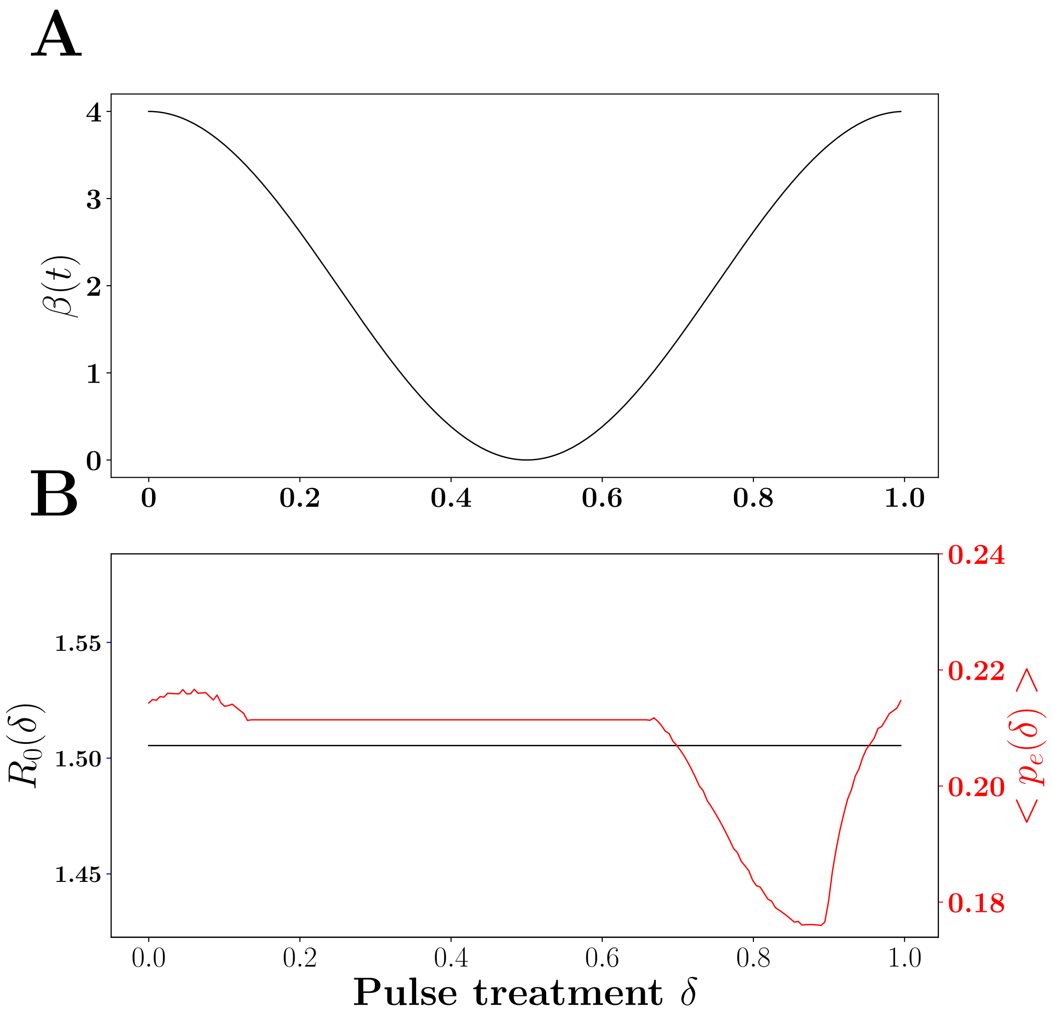

Figure H. Effect of the timing of the pulse treatment on R_0 and the mean probability of pathogen emergence. The upper curve is $\beta(t)$, pathogen transmission rate before control (figure A): it enables to identify the high and low transmission seasons. When no treatment is used the basic reproduction ratio is $R_0 = 1.505$ and the mean emergence probability is $\langle p_e \rangle = 0.302$. In figure B we plot $R_0(\delta)$, the basic reproduction number (black line), and $< p_e(\delta) >$, the mean extinction probability (red line) against δ, the timing of the pulse treatment (i.e. the amount of time after the start of the high transmission period). Parameters: $\beta = 2.0$, $\Delta t = 0.01$, $\Delta\mu = 32$.

5. **A density dependent example**

The above analysis relies on the branching process assumption where pathogen growth is not affected by density dependent effects. Yet, after some time, the spread of the pathogen is likely to reduce the density of susceptible hosts and this will feed back on the malthusian growth rate of the pathogen population. For instance, let us consider the following epidemiological model:

$$\frac{dS}{dt} = \lambda - \beta\frac{SI}{S+I} - dS \tag{5.1}$$

$$\frac{dI}{dt} = \beta\frac{SI}{S+I} - (d+\alpha)I\,. \tag{5.2}$$

Where λ is the rate at which new susceptible hosts enter the population per unit area, d is natural host death rate, α is the additional mortality induced by virulence and β is pathogen transmission rate. The above equations can be viewed as the deterministic limit of a continuous time Markov chain model that tracks the dynamics of $S^{(n)}$, the finite population size of susceptible hosts, and $I^{(n)}$, the finite population size of infected hosts. The deterministic model is the limit for $n \to \infty$ of the stochastic model for $S^{(n)}/n$, the density of the population of susceptible hosts, and $I^{(n)}/n$, the density of the population of infected hosts (see for instance Parsons et al (2018) for a derivation). When all the rates are assumed to be constant the equilibrium finite size of the population of infected hosts is equal to:

$$S_{eq}^{(n)} = \frac{n\lambda}{2d+\alpha}\,, \quad I_{eq}^{(n)} = \frac{n\lambda(d+\alpha)}{(2d+\alpha)(d+\alpha-\beta)} \tag{5.3}$$

In the following, however, all the rates are assumed constant, except β which is T-periodic:

$$\beta(t) = \beta_T(t) = \beta_0\,\mathbf{1}_{(0<t/T<1-\gamma)} \qquad (0 \le t \le T). \tag{5.4}$$

The associated branching process has periodic birth rate $\lambda(t) = \beta_T(t)$ and constant death rate $\mu = d + \alpha$. To study the effect of density dependence we performed individual based simulations to obtain the probability of emergence (the ability of the pathogen population to survive after 10 periods) after the introduction of a single infected host at time t_0. Figure I illustrates the good match between the stochastic simulations and the computation of $p_e(t_0T, T)$ under the assumption that transmission follows a square wave.

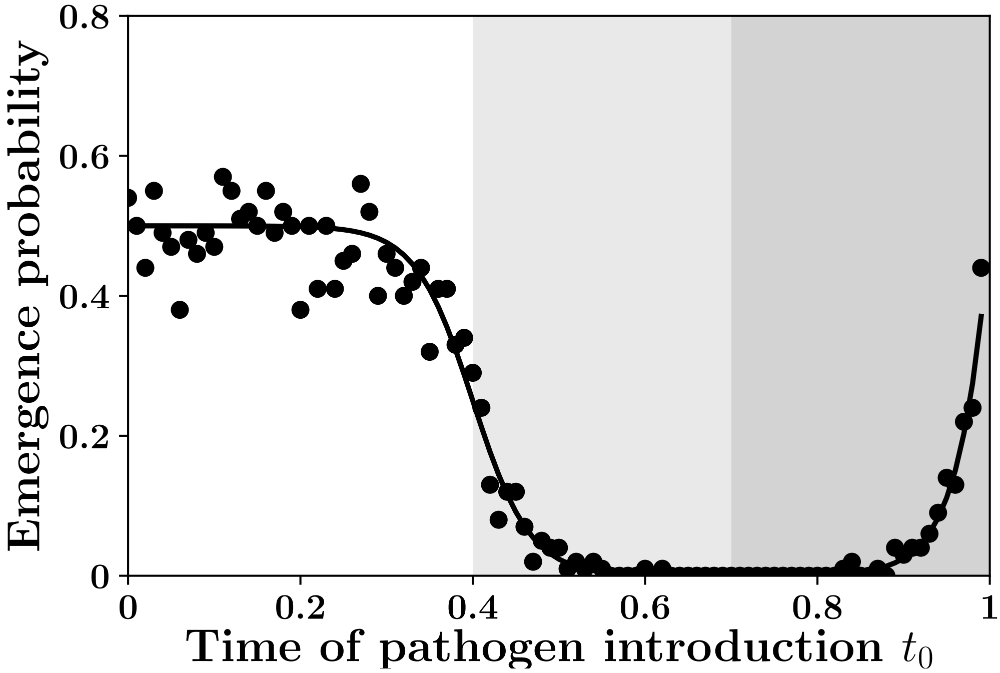

Figure I. Individual based simulations and numerical derivations of the probability of pathogen emergence. We found a good match between stochastic simulations (each black dot is obtained from 1000 replicates) and numerical derivations of the probability $p_e(t_0T, T)$ of pathogen emergence (black line). As in figures 2 (main text) and A the low transmission period is indicated by the gray shading and the *winter is coming* effect by the light gray shading. Parameters: $\beta_0 = 0.2, d = 0.09, \alpha = 0.01, \gamma = 0.3, \lambda = 1000, n = 10, T = 300$.

The absence of effect of density dependence in figure I is due to the large size of the population of infected hosts. Indeed, for the parameter values used in figure I the size of the population of infected hosts is expected to be around 52630 in the high transmission season (see equation 5.3). However, when the size of the population of infected hosts drops, the risk of pathogen extinction during the low transmission period increases. Consequently, the *winter is coming* effect is increased by density dependence. This effect can be very high and can lead the probability of pathogen emergence to zero on the whole period (see figure J).

In the following we use an heuristic argument to explain the effect of finite population size on pathogen emergence in seasonal environments. Let us focus on the the probability of emergence $p_e(0, T)$ for a pathogen introduced at time 0. If it escapes

early extinction the pathogen population will grow at a rate $r(t) > 0$ in the high transmission season. If the system size is sufficiently large, by the law of large numbers, the stochastic dynamics of $I^{(n)}(t)/n$ is very close to the deterministic solution of 5.1. So let us assume, for our heuristic, that if the infected pathogen introced at time 0 survives, at time $T(1-\gamma)$ there are exactly $I_{eq}^{(n)}$ infected hosts (see equation 5.3).
During the low transmission period $[T(1-\gamma), T]$ the number of infected hosts is a death process : infected hosts die with constant rate $\mu = d + \alpha$. Therefore, the probability that an infected survives the winter, of length γT is given by $e^{-\gamma T \mu}$ and the probability that the $I_{eq}^{(n)}$ infected hosts survive the winter is:

$$p_{winter}(n, T) = 1 - \left(1 - e^{-\gamma T(d+\alpha)}\right)^{I_{eq}^{(n)}} . \tag{5.5}$$

Hence, our heuristic gives the upper bound, $P_e(0, T) \leq p_e(0, T) p_{winter}(n, T)^{x_W}$ where x_W is the number of winters we consider, and $p_e(0, T)$ is the probability of emergence starting from zero for the associated branching process.
The curve $n \to p_{winter}(n, T)^{x_W}$ is close to a step function: close to 0 before n_c, and close to 1 after n_c, where n_c is the critical area size of the system defined by the equation: $p_{winter}(n_C, T) = 1/2$ and equals:

$$n_c = \frac{\log(1 - 2^{-1/x_W})(2d + \alpha)(\beta_0 - (d + \alpha))}{\lambda(d + \alpha)\log(1 - e^{-\gamma T(d+\alpha)})} . \tag{5.6}$$

Figure J shows results from individual based simulations (black dots) that confirm the validity of our heuristics: when $n < n_c$ the probability of pathogen emergence is droping to very low levels, and when $n > n_c$ the probability of pathogen emergence is close to $p_e(0, T)$ computed for the branching process (i.e. without density dependence).

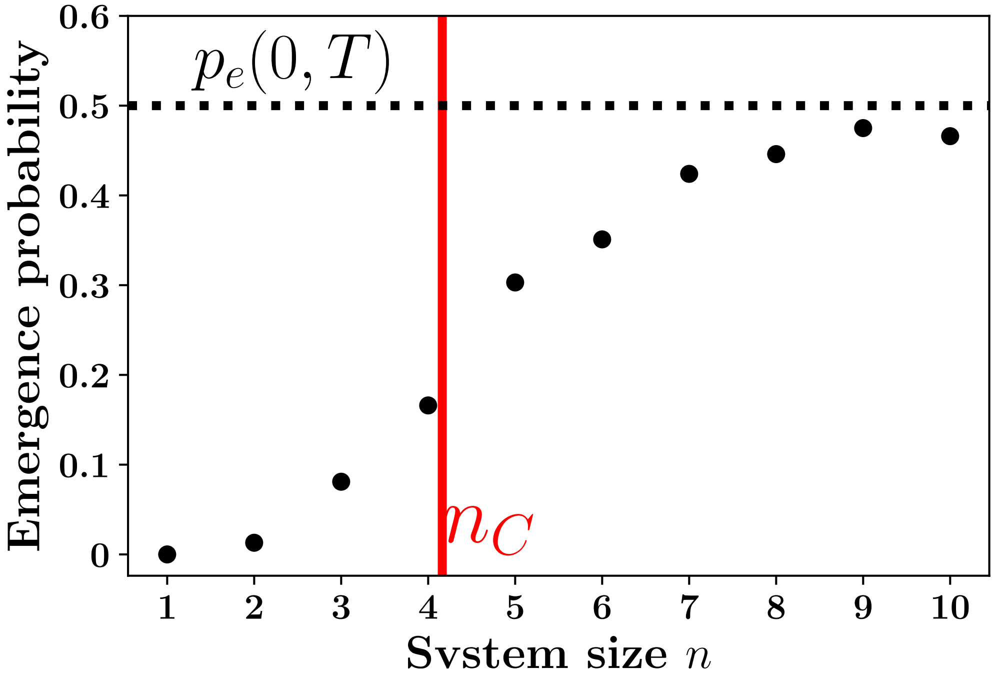

Figure J. Individual based simulations and numerical derivations of the probability of pathogen emergence $p_e(0, T)$ against the system size n. We used same parameter values as in figure I but we varied the size of the system size between $n = 1$ and 10. Our approximation for the critical system size $n_c = 4.16$ matches the point at which the probability of pathogen emergence drops. Note that our branching process approximation of the probability of pathogen emergence (i.e. $p_e = 0.5$) matches results of individual simulation when $n \gg n_c$. Parameters: $\beta_0 = 0.2, d = 0.09, \alpha = 0.01, \gamma = 0.3, \lambda = 1000, n = 10, T = 300$.

6. Additional computations and proofs

6.1. Proof of Proposition 6.1.

Proposition 6.1. *Under mild regularity assumptions on the rates λ, μ, there exists a finite set $\mathcal{F}$ such that for $t_0 \notin F$ the following limit exists $p_{e,\infty}(t_0) := \lim_{T\to+\infty} p_e(t_0T, T)$ and we have*

$$p_{e,\infty}(t_0) = \pi_e(t_0)\,\mathbf{1}_{(t_0\in A)} \tag{6.1}$$

with

$$A = \{t_0 \in [0,1]\backslash\mathcal{F}, \lambda(t_0) > \mu(t_0), \text{ and }, \forall s > 0, \varphi(t_0+s) > \varphi(t_0)\}\,.$$

The set A has the following geometric interpretation. the point $t_0 \in [0,1]$ is in A if, when the set F is empty,

- the rate function φ increases at t_0. Indeed $\varphi'(t_0) = \lambda(t_0) - \mu(t_0) > 0$.
- If you look at the future starting from t_0, that is you look at the function $s \to \varphi(t_0+s)$, you do not see a trap, that is a point lower than your starting point, that is a $s > 0$ such that $\varphi(t_0+s) \leq \varphi(t_0)$.

Another way to describe this set is the following. If t_0 is a local minimum of φ, then the points left of t_0, $s \leq t_0$ where φ is above this local minimum, that is $\varphi(s) \geq \varphi(t_0)$ are in A^C. If you do this with all the local minimums, you get the whole of A^C.

Proof. The regularity assumptions on the rate functions are the following. the functions λ and μ are assumed to be piecewise C^1, that is there exists a finite set of points $0 = x_1 < x_2 < ... < x_n = 1$ in $[0,1]$ such that λ, μ are C^1 on the segments (x_i, x_{i+1}) and $\lambda, \lambda', \mu, \mu'$ have left and right limits at the x_i's. Furthermore, we assume that for every i, the set $\{t : \varphi(t) = \varphi(x_i)\}$ is finite, with $\varphi(t) = \int_0 t(\lambda(s) - \mu(s))\,ds$. Then, Proposition 6.1 is an immediate consequence of the two well known results on Laplace integrals, which we recall here (see e.g. [9], section VII.3)
Let f be a locally bounded measurable function, and φ a non negative measurable function, both defined on $[a,b]$. For $\lambda > 0$ we let

$$F(\lambda) := \int_a^b e^{-\lambda\varphi(t)} f(t)\,dt\,. \tag{6.2}$$

If, when $t \downarrow a$, we have $f(t) = l + o(1)$ and $\varphi(t) = \alpha + (t-a)\beta + o(t-a)$ with $\beta > 0$, then

$$F(\lambda) \sim \frac{l}{\beta}\frac{1}{\lambda}e^{-\lambda\alpha} \qquad (\lambda \to +\infty). \tag{6.3}$$

(this result is thus true if f is right continous at a and φ has a positive right derivative at a).

If when $t \downarrow a$, we have $f(t) = l + o(1)$ and $\varphi(t) = \alpha + \frac{1}{2}(t-a)^2\gamma + o(t^2)$ with $\gamma > 0$, then

$$F(\lambda) \sim \sqrt{\frac{\pi}{2}} \frac{l}{\sqrt{\gamma}} \frac{1}{\sqrt{\lambda}} e^{-\lambda\alpha} \qquad (\lambda \to +\infty). \tag{6.4}$$

□

6.2. **Proof of Proposition 1.1.** Remember that we assumed that $C < \frac{R_0-1}{\lambda_0}$, that is $\varphi_\rho(1) > 0$. Forgetting about the limiting case, which we obtain by having equalities instead of inequalities, we have six cases to consider see figure K.

(1) $\varphi_\rho(t_1) < \varphi_\rho(1) < \varphi_\rho(t_2)$
(2) $\varphi_\rho(1) < \varphi_\rho(t_1) < \varphi_\rho(t_2)$
(3) $\varphi_\rho(t_2) < \varphi_\rho(t_1) < \varphi_\rho(1)$
(4) $\varphi_\rho(1) < \varphi_\rho(t_2) < \varphi_\rho(t_1)$
(5) $\varphi_\rho(t_1) < \varphi_\rho(t_2) < \varphi_\rho(1)$
(6) $\varphi_\rho(t_2) < \varphi_\rho(1) < \varphi_\rho(t_1)$

The different shapes of the integrated rate function are depicted in figure L. This figure is a companion to the proof below, since one can use this figure to spot the local minima and the corresponding t^* times using the geometric construction presented in figure A.

Finally, the actual controlled rates corresponding to the particular points of figure K are given in figure M.

Case I : $\varphi_\rho(t_1) < \varphi_\rho(1) < \varphi_\rho(t_2)$. Then we have $\langle p_{e,\infty,\rho}\rangle > \frac{R_0-1}{\lambda_0} - C = \frac{\varphi_\rho(1)}{\lambda_0}$: the strategy is not optimal.

Indeed let $t_1^*(\rho)$ be the unique $t \in (t_1, t_2)$ such that $\varphi_\rho(t_1^*(\rho)) = \varphi_\rho(1)$. Then

$$\langle p_{e,\infty,\rho}\rangle = \int_0^1 \left(1 - \frac{\mu(s)}{\lambda_\rho(s)}\right)^+ ds = \int_0^{t_1} \left(1 - \frac{1}{\lambda_0}\right) ds + \int_{t_1}^{t_1^*(\rho)} \left(1 - \frac{1}{\lambda_0(1-\rho_M)}\right) ds \tag{6.5}$$

$$= t_1\left(1 - \frac{1}{\lambda_0}\right) + (t_1^*(\rho) - t_1)\left(1 - \frac{1}{\lambda_0(1-\rho_M)}\right). \tag{6.6}$$

Since we have

$$(t_1^*(\rho) - t_1)(\lambda_0(1-\rho_M) - 1) = \varphi_\rho(t_1^*(\rho)) - \varphi_\rho(t_1) = \varphi_\rho(1) - \varphi_\rho(t_1), \tag{6.7}$$

we get that

$$\langle p_{e,\infty,\rho}\rangle = t_1\left(1 - \frac{1}{\lambda_0}\right) + \frac{\varphi_\rho(1) - \varphi_\rho(t_1)}{\lambda_0(1-\rho_M)} \tag{6.8}$$

$$= \frac{\varphi_\rho(t_1)}{\lambda_0} + \frac{\varphi_\rho(1) - \varphi_\rho(t_1)}{\lambda_0(1-\rho_M)} > \frac{\varphi_\rho(1)}{\lambda_0}, \tag{6.9}$$

since $\varphi_\rho(1) > \varphi_\rho(t_1)$.

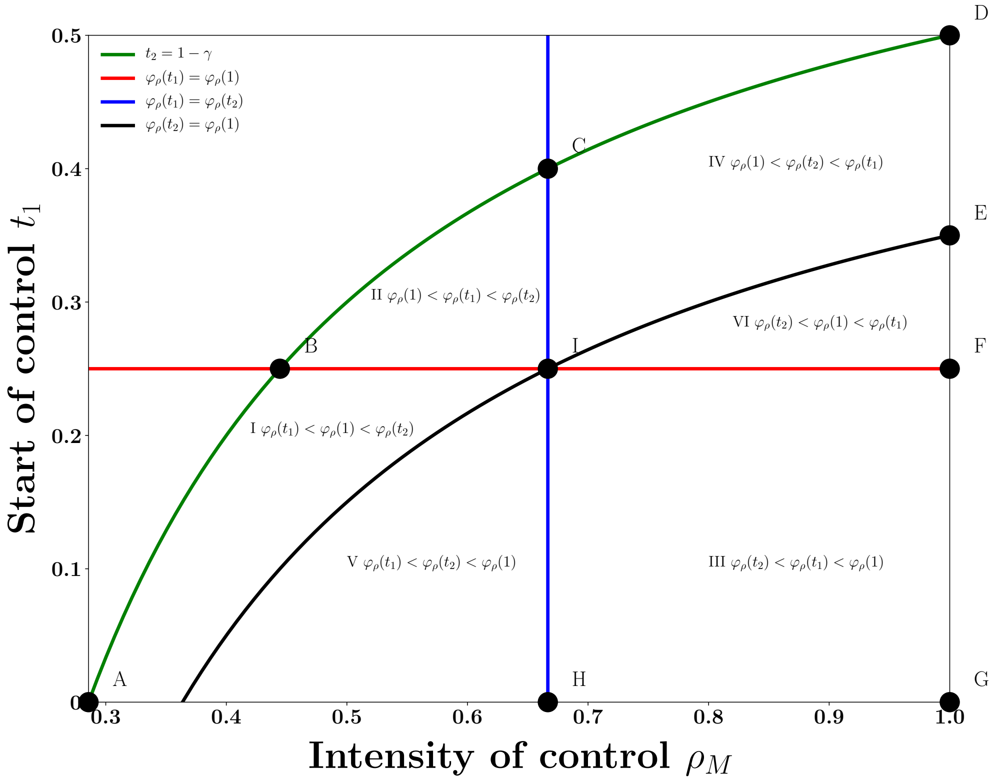

Figure K. Analysis of figure 4 of main text . We plot the six different cases (see proof of Proposition 1.1) corresponding to the respective positions of the three numbers $\varphi_\rho(t_1), \varphi_\rho(t_2), \varphi_\rho(1)$. Zones II,III,IV and VI correspond to an optimal control. The dashed red contour in figure 4 corresponds exactly to the area delineated by points B,C,D,E,F,G,H,I and B.)

Case II : $\varphi_\rho(1) < \varphi_\rho(t_1) < \varphi_\rho(t_2)$. Then we have $\langle p_{e,\infty,\rho}\rangle = \frac{\varphi_\rho(1)}{\lambda_0}$: the strategy is optimal.
Indeed let $t_1^*(\rho)$ be the unique $t \in (0, t_1)$ such that $\varphi_\rho(t_1^*(\rho)) = \varphi_\rho(1)$. Then

$$\langle p_{e,\infty,\rho}\rangle = \int_0^1 \left(1 - \frac{\mu(s)}{\lambda_\rho(s)}\right)^+ ds = \int_0^{t_1^*(\rho)} \left(1 - \frac{1}{\lambda_0}\right) ds \tag{6.10}$$

$$= t_1^*(\rho)\left(1 - \frac{1}{\lambda_0}\right) = \frac{\lambda_0 - 1}{\lambda_0} t_1^*(\rho) = \frac{\varphi_\rho(t_1^*(\rho))}{\lambda_0} = \frac{\varphi_\rho(1)}{\lambda_0}. \tag{6.11}$$

Case III : $\varphi_\rho(t_2) < \varphi_\rho(t_1) < \varphi_\rho(1)$. This is also an optimal case : $\langle p_{e,\infty,\rho}\rangle = \frac{\varphi_\rho(1)}{\lambda_0}$.
We let $t_1^*(\rho) \in (0, t_1)$ such that $\varphi_\rho(t_1^*(\rho)) = \varphi_\rho(t_2)$ and $t_2^*(\rho) \in (t_2, 1 - \gamma)$ such that $\varphi_\rho(t_2^*(\rho)) = \varphi_\rho(1)$. Then

$$\langle p_{e,\infty,\rho}\rangle = \int_0^1 \left(1-\frac{\mu(s)}{\lambda_\rho(s)}\right)^+ ds = \int_0^{t_1^*(\rho)} (1-\frac{1}{\lambda_0})\,ds + \int_{t_2}^{t_2^*(\rho)} (1-\frac{1}{\lambda_0})\,ds \quad (6.12)$$

$$= (1-\frac{1}{\lambda_0})(t_1^*(\rho) + (t_2 - t_2^*(\rho)))\,. \quad (6.13)$$

Since we have

$$\varphi_\rho(t_1^*(\rho)) = (\lambda_0 - 1)t_1^*(\rho) = \varphi_\rho(t_2) \quad (6.14)$$

$$\varphi_\rho(t_2^*(\rho)) - \varphi_\rho(t_2) = \varphi_\rho(1) - \varphi_\rho(t_2) = (t_2^*(\rho) - t_2)(\lambda_0 - 1)\,, \quad (6.15)$$

we get that

$$\langle p_{e,\infty,\rho}\rangle = \frac{1}{\lambda_0}(\varphi_\rho(t_2) + \varphi_\rho(1) - \varphi_\rho(t_2)) = \frac{\varphi_\rho(1)}{\lambda_0}\,. \quad (6.16)$$

Case IV: $\varphi_\rho(1) < \varphi_\rho(t_2) < \varphi_\rho(t_1)$. This is also an optimal case : $\langle p_{e,\infty,\rho}\rangle = \frac{\varphi_\rho(1)}{\lambda_0}$. We let $t_1^*(\rho) \in (0, t_1)$ such that $\varphi_\rho(t_1^*(\rho)) = \varphi_\rho(1)$ and we get

$$\langle p_{e,\infty,\rho}\rangle = \int_0^1 \left(1-\frac{\mu(s)}{\lambda_\rho(s)}\right)^+ ds = \int_0^{t_1^*(\rho)} (1-\frac{1}{\lambda_0})\,ds = t_1^*(\rho)(1-\frac{1}{\lambda_0}) \quad (6.17)$$

$$= \frac{1}{\lambda_0}(\lambda_0 - 1)t_1^*(\rho) = \frac{1}{\lambda_0}\varphi_\rho(t_1^*(\rho)) = \frac{\varphi_\rho(1)}{\lambda_0} \quad (6.18)$$

Case V :$\varphi_\rho(t_1) < \varphi_\rho(t_2) < \varphi_\rho(1)$. The strategy is not optimal.
Let $t_2^*(\rho) \in (t_2, 1)$ such that $\varphi_\rho(t_2^*(\rho)) = \varphi_\rho(1)$. Then

$$\langle p_{e,\infty,\rho}\rangle = \int_0^{t_2^*(\rho)} (1-\frac{1}{\lambda_\rho(s)})\,ds \quad (6.19)$$

$$= (1-\frac{1}{\lambda_0})(t_1 + t_2^*(\rho) - t_2) + (1-\frac{1}{\lambda_0(1-\rho_M)})(t_2 - t_1) \quad (6.20)$$

$$= \frac{\varphi_\rho(t_2^*(\rho)) - \varphi_\rho(t_2) + \varphi_\rho(t_1)}{\lambda_0} + \frac{\varphi_\rho(t_2) - \varphi_\rho(t_1)}{\lambda_0(1-\rho_M)} \quad (6.21)$$

$$= \frac{\varphi_\rho(1) - \varphi_\rho(t_2) + \varphi_\rho(t_1)}{\lambda_0} + \frac{\varphi_\rho(t_2) - \varphi_\rho(t_1)}{\lambda_0(1-\rho_M)} \quad (6.22)$$

$$= \frac{1}{\lambda_0}(\varphi_\rho(1) + (\varphi_\rho(t_2) - \varphi_\rho(t_1))\frac{\rho_M}{1-\rho_M} > \frac{1}{\lambda_0}\varphi_\rho(1)\,. \quad (6.23)$$

Case VI: $\varphi_\rho(t_2) < \varphi_\rho(1) < \varphi_\rho(t_1)$. This is an optimal strategy.

Let $t_1^*(\rho) \in (0, t_1)$ such that $\varphi_\rho(t_1^*(\rho)) = \varphi_\rho(1)$. Then

$$\langle p_{e,\infty,\rho} \rangle = \int_0^{t_1^*(\rho)} (1 - \frac{1}{\lambda_0}) ds \tag{6.24}$$

$$= \frac{1}{\lambda_0}(\lambda_0 - 1) t_1^*(\rho) = \frac{1}{\lambda_0} \varphi_\rho(t_1^*(\rho)) \tag{6.25}$$

$$= \frac{1}{\lambda_0} \varphi_\rho(1). \tag{6.26}$$

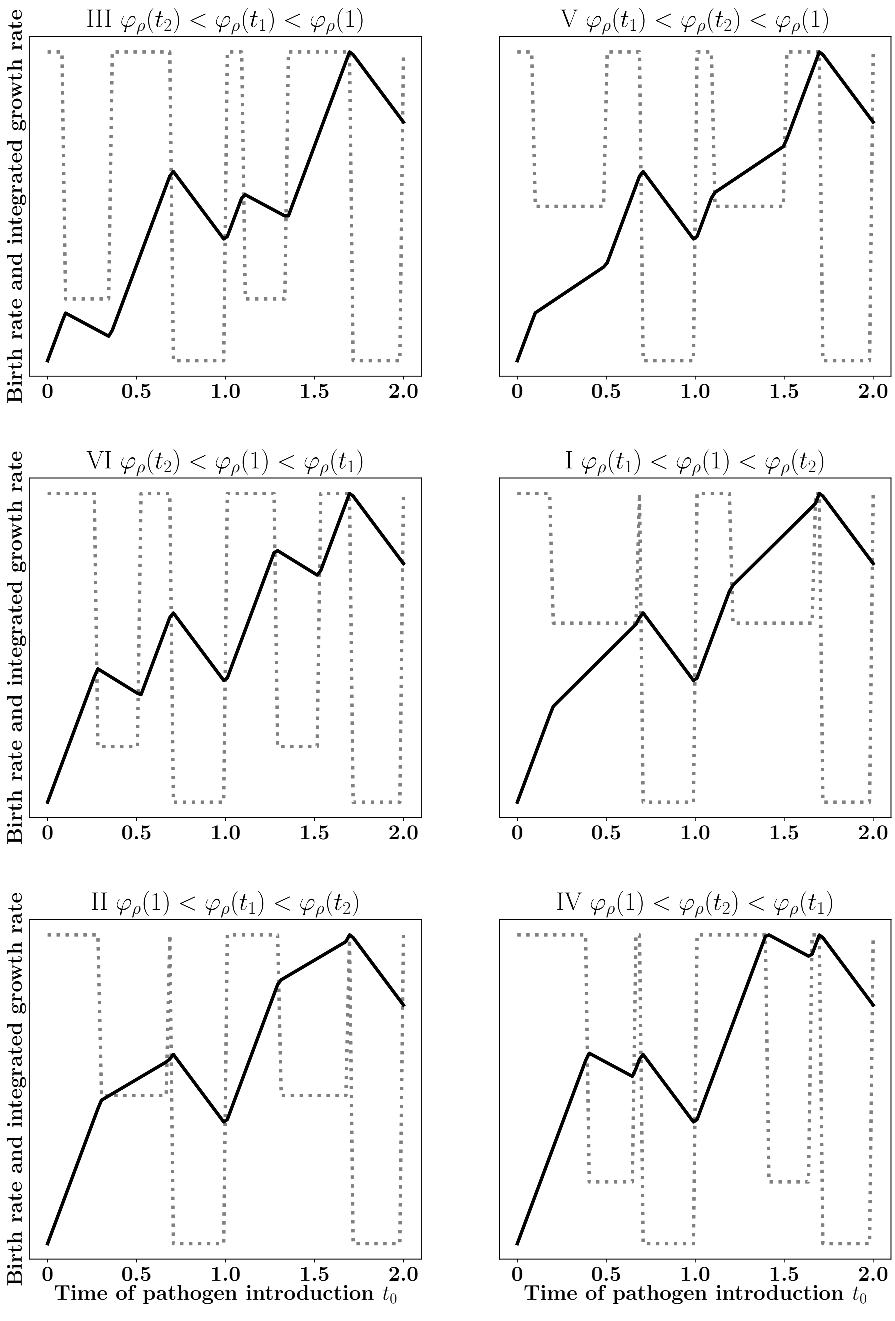

Figure L. Understanding the different cases in figure K. The dotted black line is the controlled birth rate $\lambda_\rho(t)$, the solid black line is the controlled integrated growth rate $\varphi_\rho(t)$.

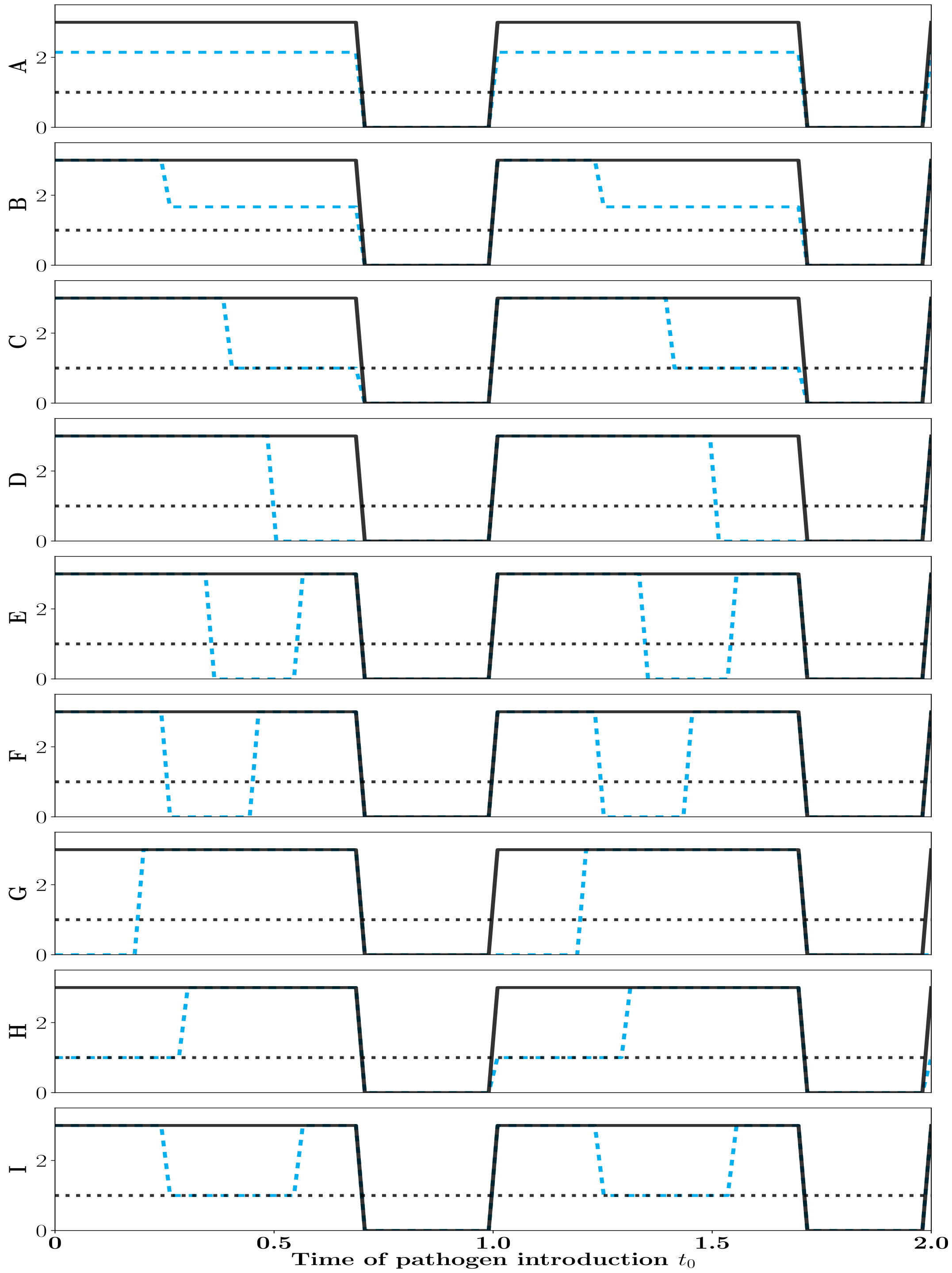

Figure M. Understanding the 9 different points in figure K. The solid black line is the uncontrolled birth rate $\lambda(t) = \lambda_0 \mathbf{1}_{(0<t<1-\gamma)}$, the dashed blue line is the controlled birth rate $\lambda_\rho(t)$, the dotted black line is the death rate $\mu = 1$. Parameters are $\lambda_0 = 3.0, \gamma = 0.3, C = 0.2$.

References

1. Bacaër N, Guernaoui S. The epidemic threshold of vector-borne diseases with seasonality. The case of cutaneous leishmaniasis in Chichaoua, Morocco. J Math Biol. 2006;53(3):421–436. doi:10.1007/s00285-006-0015-0.
2. Bacaër N, Ait Dads EH. On the probability of extinction in a periodic environment. J Math Biol. 2014;68(3):533–548. doi:10.1007/s00285-012-0623-9.
3. Lourenço J, Maia de Lima M, Faria NR, Walker A, Kraemer MU, Villabona-Arenas CJ, et al. Epidemiological and ecological determinants of Zika virus transmission in an urban setting. eLife. 2017;6:e29820. doi:10.7554/eLife.29820.
4. Suparit P, Wiratsudakul A, Modchang C. A mathematical model for Zika virus transmission dynamics with a time-dependent mosquito biting rate. Theoretical Biology and Medical Modelling. 2018;15(1). doi:10.1186/s12976-018-0083-z.
5. Zhang Q, Sun K, Chinazzi M, Pastore y Piontti A, Dean NE, Rojas DP, et al. Spread of Zika virus in the Americas. Proceedings of the National Academy of Sciences. 2017;114(22):E4334–E4343. doi:10.1073/pnas.1620161114.
6. Lambrechts L, Paaijmans KP, Fansiri T, Carrington LB, Kramer LD, Thomas MB, et al. Impact of daily temperature fluctuations on dengue virus transmission by Aedes aegypti. Proceedings of the National Academy of Sciences. 2011;108(18):7460–7465. doi:10.1073/pnas.1101377108.
7. Grassly NC, Fraser C. Seasonal infectious disease epidemiology. Proceedings Biological sciences, The Royal Society. 2006;273:2541–2550. doi:10.1098/rspb.2006.3604.
8. Kendall DG. On the generalized "birth-and-death" process. Ann Math Statistics. 1948;19:1–15. doi:10.1214/aoms/1177730285.
9. Faraut J. Calcul intégral. Collection Enseignement sup. Mathématiques. EDP Sciences; 2006. Available from: `https://books.google.fr/books?id=DtE0aBG9lVYC`.

P. Carmona, Laboratoire de Mathématiques Jean Leray, Université de Nantes, 2 Rue de la Houssinière, F-44322 Nantes Cedex
Email address: `philippe.carmona@univ-nantes.fr`

Sylvain Gandon, Centre d'Écologie Fonctionnelle et Évolutive, UMR 5175 C.N.R.S, 1919 Route De Mende, 34293 Montpellier 5
Email address: `Sylvain.Gandon@cefe.cnrs.fr`
