# Supplementary material for: Winter is coming: Pathogen emergence in seasonal environments
Source: PLoS Comput Biol. 2020 Jul 6;16(7):e1007954. doi: 10.1371/journal.pcbi.1007954 (PMC7365480; doi:10.1371/journal.pcbi.1007954)
Supplement: Attachment — Submitted filename: Review_Seasonality.pdf [file pcbi.1007954.s002.pdf]

This is a challenging paper which makes an important contribution to the study of seasonally forced systems. I enjoyed reading the paper but it took me a while to get my head around many of the ideas presented.

I suggest the authors try and make their work more approachable by enhancing the presentation. The figures are very helpful, but they are only fleetingly explained in the text, so the reader is forced to untangle the ideas from a study of the figure legends. It would help considerably if the ideas in the figure legends are integrated better into the main text.

Even the main "Winter is coming effect" cannot be easily understood without reference to the figures, which cannot be easily understood without focused concentrated reading of the figure legends. Thus the reader has to work through a series of almost annoying disruptions before really appreciating the main point.

Some equations in the main text appear out of the blue with unknown derivation. It's important to mention that their derivation may be found in the Methods or SI.

In addition to the examples mentioned here, the authors could add further effort into simplifying the paper wherever possible, expanding some sections if need be.

Some comments, mostly minor:

L.16 "Under the reasonable assumption that the initial spread of directly transmitted disease follows a one dimensional birth-death branching process the probability of emergence is zero when $R0 < 1$ and, when $R0 > 1$,"

The authors should refer to the stochastic threshold theorem of Whittle discussed in eg Renshaw, and perhaps elaborate with another sentence or two

L.31 Eqn.2 I do not have easy access to the references that are the basis for your key eqn.2. It could be useful to sketch the derivation in the SI, since its such an important equation for the paper.

Line 32: malthusian growth rate → Malthusian

L40: In this case the probability of emergence can also be expressed as a ratio of average birth and death rates, but with different weights:….."

It would seriously help to state that you derive this (eqn.4) in the Methods and its not plucked out of thin air.

L 60: "we start our analysis with a directly transmitted disease with a constant duration of infection, $u(t) = u$, but…."

$u(t)$ is not the duration of infection

The seasonality set up has some commonalities to the work of Stone, Olinky &Huppert "Seasonal dynamics of recurrent epidemics." Nature 2007.

L.103 "For a given investment in disease control C, what are the values of t1, t2 and _M that minimize the average probability of emergence pei? We first answer this question when the fluctuation of transmission is a square wave where _(t) oscillates between … throughout the year (Figure 3). "

I didn't see an answer to this question, apart from numerical simulations which were not really discussed here.

Fig.2 is hardly explained in main text, yet it is of considerable importance to understanding the key concept.

L.141 Eqn.7 Please explain/define the key terms in the formula

L.197 "Indeed, we found that the probability of emergence is minimized if pulse vaccination occurs a bit sooner than the time at which R0 is minimized (3.71 instead of…"

I'm not sure this was mentioned previously. Perhaps the authors could tell the reader that "an examination of figure * shows that the probability of emergence is minimized if …".

L.257 "the basic reproduction number is R0 = _ and the probability of extinction, starting initially with one individual, is q = inf(1; 1/ R0) (Figure 1). "

This could be related to stochastic threshold theorem mentioned above.

L.303 "A demographic trap occurs if there is a time s > t0 for which the expected size of the population is smaller than the original size at the introduction time :
E[X(s) | X(t0) = x0] = ….. (28) "

I may be wrong, but I don't think X(s) is defined here or in the paper.

SI. Perhaps explain the vector notation below. What is alpha?

1.1. **Square wave.** We assume that death rate is constant $\mu(t) = 1$ and $\lambda(t) = \lambda_0 \; \mathbf{1}_{(0<t<1-\gamma)}$ with $\lambda_0 > 1$. Assume furthermore that $R_0 > 1$ that is $\bar{\lambda} > \bar{\mu}$ with $\bar{\lambda} = \int_0^1 \lambda(s)\, ds = \lambda_0(1 - \gamma + \alpha\gamma)$ and $\bar{\mu} = 1$.

[illegible]
