# Supplementary material for: Winter is coming: Pathogen emergence in seasonal environments
Source: PLoS Comput Biol. 2020 Jul 6;16(7):e1007954. doi: 10.1371/journal.pcbi.1007954 (PMC7365480; doi:10.1371/journal.pcbi.1007954)
Supplement: Attachment — Submitted filename: CoverLetter-ResponseToReviewers.pdf [file pcbi.1007954.s003.pdf]

We thank the reviewer for very helpful comments, which have helped to improve our manuscript. We explain below how we tried to address all of those points in the revised version of our manuscript. All the modifications are indicated in blue in the main text and in the Supplementary Information.

This is a challenging paper which makes an important contribution to the study of seasonally forced systems. I enjoyed reading the paper but it took me a while to get my head around many of the ideas presented.

I suggest the authors try and make their work more approachable by enhancing the presentation. The figures are very helpful, but they are only fleetingly explained in the text, so the reader is forced to untangle the ideas from a study of the figure legends. It would help considerably if the ideas in the figure legends are integrated better into the main text.

Even the main "Winter is coming effect" cannot be easily understood without reference to the figures, which cannot be easily understood without focused concentrated reading of the figure legends. Thus the reader has to work through a series of almost annoying disruptions before really appreciating the main point.

Thank you for these constructive comments. We extended the description of the results presented in our figures in the revised version of the manuscript. We believe this improved the accessibility of our main results.

Some equations in the main text appear out of the blue with unknown derivation. It's important to mention that their derivation may be found in the Methods or SI.

We added more explicit references to the SI to be more explicit about where to find the derivation of the different results presented in the main text.

In addition to the examples mentioned here, the authors could add further effort into simplifying the paper wherever possible, expanding some sections if need be.

Some comments, mostly minor:

L.16 "Under the reasonable assumption that the initial spread of directly transmitted disease follows a one dimensional birth-death branching process the probability of emergence is zero when $R0 < 1$ and, when $R0 > 1$,"

The authors should refer to the stochastic threshold theorem of Whittle discussed in eg Renshaw, and perhaps elaborate with another sentence or two

This is indeed a very classical result and we now refer more explicitly to earlier studies on stochastic epidemics (Bailey 1953, Whittle1955).

L.31 Eqn.2 I do not have easy access to the references that are the basis for your key eqn.2. It could be useful to sketch the derivation in the SI, since its such an important equation for the paper.

We explicitly relate to the section of the SI and the methods.

Line 32: malthusian growth rate → Malthusian

Modified in the revised version.

L40: In this case the probability of emergence can also be expressed as a ratio of average birth and death rates, but with different weights:…..”

It would seriously help to state that you derive this (eqn.4) in the Methods and its not plucked out of thin air.

We explicitly relate to the section of the SI and the methods.

L 60: “we start our analysis with a directly transmitted disease with a constant duration of infection, u(t) = u, but….”

u(t) is not the duration of infection

Thanks. We meant “constant rate of clearance”. The duration of infection is exponentially distributed in our model. We modified the revised version of the manuscript accordingly.

The seasonality set up has some commonalities to the work of Stone, Olinky &Huppert “Seasonal dynamics of recurrent epidemics.” Nature 2007.

Thank you very much for pointing out this reference. This work is indeed relevant to the *winter is coming* effect since it clearly shows a link between the timing (month) and the size of the epidemic. In particular, small epidemics are always occurring at the end of the “high transmission” season. We discuss that in at the end of the revised version (lines 205-209)

L.103 “For a given investment in disease control C, what are the values of t1, t2 and _M that minimize the average probability of emergence pei? We first answer this question when the fluctuation of transmission is a square wave where _(t) oscillates between … throughout the year (Figure 3). “

I didn’t see an answer to this question, apart from numerical simulations which were not really discussed here.

We extended our presentation of figures 3 and 4 in the main text (lines 120-132). These explanations help grasp the main results of the figures without having to read the figure legends.

Fig.2 is hardly explained in main text, yet it is of considerable importance to understanding the key concept.

We have extended our description of the results of Figure 2. In particular we present the geometric argument for the “winter is coming” effect in the main text (lines 90-98).

L.141 Eqn.7 Please explain/define the key terms in the formula

We developed the terms of this expression and we provide an intuitive interpretation of these quantities (lines 160-171).

L.197 "Indeed, we found that the probability of emergence is minimized if pulse vaccination occurs a bit sooner than the time at which R0 is minimized (3.71 instead of…"

I'm not sure this was mentioned previously. Perhaps the authors could tell the reader that "an examination of figure * shows that the probability of emergence is minimized if …".

Modified accordingly.

L.257 "the basic reproduction number is R0 = _ and the probability of extinction, starting initially with one individual, is q = inf(1; 1/ R0) (Figure 1). "

This could be related to stochastic threshold theorem mentioned above.

This is indeed a classical result and we added a reference to the work of Whittle.

L.303 "A demographic trap occurs if there is a time s > t0 for which the expected size of the population is smaller than the original size at the introduction time :
E[X(s) | X(t0) = x0] = ….. (28) "

I may be wrong, but I don't think X(s) is defined here or in the paper.

We clarified this in the revised version (X(s) refers to the epidemic size).

SI. Perhaps explain the vector notation below. What is alpha?

Thanks there was a typo in this equation. We corrected the SI accordingly.
