# Supplementary material for: Winter is coming: Pathogen emergence in seasonal environments
Source: PLoS Comput Biol. 2020 Jul 6;16(7):e1007954. doi: 10.1371/journal.pcbi.1007954 (PMC7365480; doi:10.1371/journal.pcbi.1007954)
Supplement: Attachment — Submitted filename: CoverLetter_Revision2.pdf [file pcbi.1007954.s004.pdf]

We thank the two reviewers for very helpful comments. We explain below how we addressed all of those points in the revised version of our manuscript.

As with all papers reviewed by the journal, your manuscript was reviewed by members of the editorial board and by two referees. The first referee had commented on the previous version of the manuscript and had requested clarifications and changes to make the work more accessible. She/he is now satisfied with the changes and provides minor suggestions.

We incorporated all these minor suggestions in the revised version.

The second referee agrees that the work is a valuable and interesting contribution. But his/her major concerns are again with the way the work is presented, in particular the lack of sufficient context motivating the work and placing its relevance into a more tangible and historical context. Specific suggestions are made for the consideration of a number of studies that would help doing this. We happen to agree that the manuscript can be written in a way to reach a broader audience and goes from the technical contribution to deeper context and discussion.

As detailed below we referred to some of these earlier studies. Some of these papers are indeed very relevant. Other papers, however, are less distantly related to our work. We also have to recall that our approach is based on the calculation of the probability of non-extinction after several seasons. In contrast, some of the studies are only discussing outcomes in a single season.

In light of this review (below this email) and our own assessment, we would like to invite the resubmission of a revised version that takes into account the reviewer's comments in a way that makes the results of the work more compelling. We cannot make any decision about publication until we have seen the revised manuscript.

We incorporated all these comments and hop the revised version will be suitable for publication in PLoS Comp Biol.

***

Reviwer#1

This is a very carefully worked out and mature work that covers a lot of ground from periodically forced natural systems, to their optimal control. The authors have a detailed knowledge of stochastic systems and branching processes, and this has led to the fruition of a deep analysis from many fronts. I learnt a lot from reading this manuscript.

1) The authors have made significant new contributions to the study of forced seasonal systems and highlighted some new (sometimes subtle) important dynamical behavious.

2) I have checked all the maths in the SI that is relevant to the equations and models discussed in the main text.

Thank you for these positive comments.

Minor comments:

*Authors should mention that Eqns 5&6 are derived in the relevant Appendix.

Done. These results are derived in section 2 of the Methods section.

*Page 5 Eqn.5 Perhaps give the value of R0, and pE in the main text, for Fig.2E and 2F so the reader can see this works.

We chose to develop the figure legend of figure 2 to highlight the good match between these approximations and the numerical results.

*Ref missing in Section 2.3 of SI

Done.

*Fig.2 The notation p(toT, T) is used in the SI, but I don't think it is given in the main text. (I am referring here to the rescaling of t0)

The notation p(toT,T) is used in the methods section but also in the figure 1 which appears in the main text. We provide a bit more explanations in the methods about this rescaling (lines 319).

***

Reviewer #2: The topic and idea developed in this work is very interesting, and I find it particularly valuable that the authors emphasize the importance of working with probabilities rather than with deterministic quantities such as R0. Although R0 is useful for many purposes, it presents limitations, especially for low-transmission situations, among others.

Although the development of expressions and the study of probabilities for the occurrence of major outbreaks are not new, there is a lack of concrete applications and discussion of different transmission scenarios. This theoretical study investigates how the interaction of seasonality, duration and arrival time of the infection, affects the probability of a major outbreak. In particular, the authors contribute to the formalization of an effect that is somehow intuitive which they call “the winter is coming effect”.

Thank you for these positive comments.

Unfortunately, however, the authors do not better exploit the intuitive and biological aspects of this effect in a way that would be accessible to a broader audience (that would be very interested in this topic). The way the paper is written is quite technical. It would benefit from a deeper connection to particular infectious diseases or actual transmission results, mainly in the introduction and discussion sections. For example, the authors could compare/connect their results with Otero et al. 2010, who studied the probabilities of major dengue outbreaks based on the first infection arrival time to the city of Buenos Aires.

We refer to this interesting simulation study in line 212 when we discuss the effect of the timing of the introduction on epidemic size. Note, however, that the probability of emergence in this study is not equivalent to our analysis because they focus on a single season. As discussed in our paper, an epidemic could occur the first year and get extinct during the winter season. This is particularly true for epidemics that start late and induce a small number of cases. The winter is coming effect is driven by the interplay between epidemic size just before winter and the probability to survive throughout the winter.

Another example that could be discussed in this paper is the study of flu arrival times for different cities in the US and UK by Truscott & Ferguson 2012, as well as the results obtained by Dalziel et al. 2018 also for flu.

These two papers are focusing on the timing of the start of epidemics in spatially structured environments. It is loosely connected to what we are discussing and we are concerned that trying to discuss this connection could be a bit confusing. Our discussion is already quite long and covers many different biological examples that are closer to the model we analyse.

In addition, I was surprised that the authors did not cite and connect their probabilities' expressions with those in the papers by Bartlett 1964 and by Lloyd et. al 2007. Both papers discuss the probability of pathogen emergence, in direct and vector transmitted diseases.

Thank you for pointing this out. These two papers are really useful and we refer to them explicitly several times in our revised version.

In conclusion, I like the idea and I think it is a compelling topic that a broad spectrum of readers of this journal and beyond would be interested in. The writing is too technical and needs deeper motivation and discussion related to empirical and intuitive aspects, including a clearer connection to results in other studies of transmissible diseases.

Some parts of the paper are necessarily technical but the abstract, the author summary, the introduction and the discussion are written for biologists. We provide many intuitive explanations for our results. We relate to some empirical evidence but we do not want to introduce confusion by discussing loosely connected papers. Emergence is a subtle concept because it refers to long term persistence after a single introduction event. It is difficult to obtain good empirical or experimental data on this. Yet, it is a key concept if one wants to optimize control strategies.
